# Identification of an O-antigen chain length regulator, WzzP, in *Porphyromonas gingivalis*

**DOI:** 10.1002/mbo3.84

**Published:** 2013-03-19

**Authors:** Mikio Shoji, Hideharu Yukitake, Keiko Sato, Yasuko Shibata, Mariko Naito, Joseph Aduse-Opoku, Yoshimitsu Abiko, Michael A Curtis, Koji Nakayama

**Affiliations:** 1 Division of Microbiology and Oral Infection, Department of Molecular Microbiology and Immunology, Nagasaki University Graduate School of Biomedical SciencesNagasaki, 852-8588, Japan; 2 Department of Biochemistry and Molecular Biology, Nihon University School of Dentistry at MatsudoChiba, 271-8587, Japan; 3 Centre for Immunology and Infectious Disease, Blizard Institute, Barts and the London School of Medicine and Dentistry, Queen Mary University of LondonLondon, E1 2AT, United Kingdom; 4 Global COE Program at Nagasaki UniversityNagasaki, Japan

**Keywords:** LPS biosynthesis, periodontal pathogen, WbaP protein, Wzx protein, Wzz protein

## Abstract

The periodontal pathogen *Porphyromonas gingivalis* has two different lipopolysaccharides (LPSs) designated O-LPS and A-LPS, which are a conventional O-antigen polysaccharide and an anionic polysaccharide that are both linked to lipid A-cores, respectively. However, the precise mechanisms of LPS biosynthesis remain to be determined. In this study, we isolated a pigment-less mutant by transposon mutagenesis and identified that the transposon was inserted into the coding sequence PGN_2005, which encodes a hypothetical protein of *P. gingivalis* ATCC 33277. We found that (i) LPSs purified from the PGN_2005 mutant were shorter than those of the wild type; (ii) the PGN_2005 protein was located in the inner membrane fraction; and (iii) the PGN_2005 gene conferred Wzz activity upon an *Escherichia coli wzz* mutant. These results indicate that the PGN_2005 protein, which was designated WzzP, is a functional homolog of the Wzz protein in *P. gingivalis*. Comparison of amino acid sequences among WzzP and conventional Wzz proteins indicated that WzzP had an additional fragment at the C-terminal region. In addition, we determined that the PGN_1896 and PGN_1233 proteins and the PGN_1033 protein appear to be WbaP homolog proteins and a Wzx homolog protein involved in LPS biosynthesis, respectively.

## Introduction

*Porphyromonas gingivalis* is a Gram-negative anaerobic bacterium considered a major etiological agent in chronic periodontitis (Haffajee and Socransky 1994) and may be associated with systemic conditions, such as cardiovascular diseases (Pussinen and Mattila 2004), preterm low birth weight (Madianos et al. 2001), and rheumatoid arthritis (Lundberg et al. 2010). The surface components of *P. gingivalis*, such as the major cell surface macromolecules including exopolysaccharide (EPS), lipopolysaccharide (LPS), and capsular polysaccharide (CPS) (or K-antigen) and the extracellular/surface cysteine proteases including Arg-gingipain (Rgp) and Lys-gingipain (Kgp), are considered important virulence factors in the pathogenesis of periodontal diseases (Lamont and Jenkinson 1998).

For many bacteria, surface polysaccharides play critical roles in immune modulation and evasion (Comstock and Kasper 2006). As surface polysaccharides constitute the outer layer of the outer membrane and form a defensive barrier against the host's immune system, their highly antigenic variations can lead to strain-specific properties. *Porphyromonas gingivalis* has two distinct polysaccharides on the cell surface: LPSs and CPSs (Arndt and Davey 2010). LPS consists of three general components: O-antigen polysaccharide, core oligosaccharide, and lipid A. Paramonov et al. (2001) demonstrated that the O-antigen of *P. gingivalis* strain W50 consists of a tetrasaccharide repeating unit composed of (6)-α-d-Glc*p*-(1-4)-α-l-Rha*p*-(1-3)-β-d-GalNAc-(1-3)-α-d-Gal*p*-(1). In addition, it was recently shown that *P. gingivalis* synthesizes another surface polysaccharide, which is distinct from O-LPS and capsular polysaccharide (Paramonov et al. 2005; Aduse-Opoku et al. 2006; Rangarajan et al. 2008). Initially, the anionic polysaccharide (APS) was thought to be associated with the cell envelope through an unknown mechanism. As the APS was found to be anchored to the cell surface by lipid A, it was categorized as an LPS molecule and designated A-LPS (Rangarajan et al. 2008). Curtis et al. (1999) obtained a monoclonal antibody (mAb 1B5) that was originally raised against the catalytic domain of RgpA protease. The mAb 1B5 cross-reacts with A-LPS and recognizes a phosphorylated branched mannan in the APS repeating unit (Paramonov et al. 2005).

Our previous study indicated that a *porR* gene encoding a putative aminotransferase plays a role in colonial pigmentation on blood agar plates. A *porR* mutant presented a decrease of cell-associated Rgp and Kgp activities and no reduction of secreted Rgp or Kgp activity, and the mAb 1B5 did not recognize any products of the *porR* mutant (Shoji et al. 2002). Furthermore, mutants of *vimA*, *vimE*, *vimF* (Vanterpool et al. 2005a,b), *wbpB* (Slaney et al. 2006), *rfa* (Sato et al. 2009), *waaL* (Rangarajan et al. 2008), *wzy* (Paramonov et al. 2009), *gtfB* (Yamaguchi et al. 2010), PGN_0242 and PGN_0663 (Shoji et al. 2011) also exhibited no immunoreaction to mAb 1B5, indicating that these genes as well as *porR* are involved in the A-LPS biosynthesis. *porR* and *wbpB* are predicted to be involved in the initial synthesis of structural sugar(s) within APS. The *rfa* gene is thought to be involved in the synthesis of the core oligosaccharide of LPS (Sato et al. 2009). The *vimA*, *vimE*, and *vimF* genes play a role in the regulation of gingipain activities, but their precise roles in A-LPS biosynthesis are still unknown. Further study is therefore needed to identify the other factors that are required for the biosynthesis of both A-LPS and O-LPS.

The study of *porR* indicated that gingipains and hemagglutinin proteins are linked to the A-LPS, suggesting that A-LPS plays a critical role in the anchorage of cell surface virulence factors (Shoji et al. 2002). The gingipains and hemagglutinin proteins possess a conserved C-terminal domain (CTD) in their primary sequences. We recently demonstrated that CTD-containing proteins are secreted onto the cell surface via the Por secretion system (PorSS)/Type IX secretion system (T9SS) (Sato et al. 2010; Shoji et al. 2011; Sato et al. 2013; McBride and Zhu 2013). Among the CTD proteins, RgpB (Nguyen et al. 2007), TapA (Kondo et al. 2010), HBP35 (Shoji et al. 2010, 2011), and CPG70 (Chen et al. 2011) have been shown to form diffuse bands on an SDS-PAGE (sodium dodecyl sulfate polyacrylamid gel electrophoresis) gel, suggesting that they are linked to A-LPS. To understand the pathogenesis of *P. gingivalis*, it is important to analyze not only A-LPS biosynthesis but also the glycosylated form of CTD proteins.

In LPS biosynthesis pathways, the lipid A-core and the O-antigen are independently assembled on the cytoplasmic side of the inner membrane. Subsequently, these molecules are separately translocated to the periplasmic side of the inner membrane. Next, the lipid A-core and the O-antigen are joined covalently by the O-antigen ligase, WaaL. The mature LPS is transported onto the cell surface by the LPS transport proteins (Sperandeo et al. 2009). Three steps that are important for LPS biosynthesis are completed by the inner membrane proteins. First, the initiating enzyme, such as WbaP or WecA, links a sugar residue with one phosphate onto an undecaprenyl monophosphate (UndP) at the cytoplasmic side of the inner membrane. Then, further sugar residues are sequentially added by glycosyl transferases using nucleotide-activated sugars as substrates. After UndPP-glycans are assembled, these are transported either via the Wzx-like flippases or the adenosine triphosphate (ATP)-binding cassette transporters (ABC transporters), such as the Wzt and Wzm proteins. Finally, polysaccharides from the UndPP carrier, which are polymerized by Wzy and Wzz proteins, are transferred on terminal sugar residues of the lipid A-core by an O-antigen ligase, WaaL. It has been shown that PGN_1242 and PGN_1302 correspond to the Wzy and WaaL proteins, respectively (Rangarajan et al. 2008; Paramonov et al. 2009; Haurat et al. 2011). However, other responsible genes encoding initiation enzymes for UndP, UndPP glycans transporters, or a Wzz protein that is critical for LPS biosynthesis have yet to be identified.

In this study, we found that the *P. gingivalis* PGN_2005 protein is a functional homolog of the Wzz protein. Our results also suggest that the PGN_1896 and PGN_1233 proteins and the PGN_1033 protein play roles as, respectively, the WbaP homolog proteins and the Wzx homolog protein involved in *P. gingivalis* LPS biosynthesis.

## Experimental Procedures

### Bacterial strains and plasmids

The bacterial strains and plasmids used in this study are listed in Tables S2 and S3, respectively.

### Media and conditions for bacterial growth

*Porphyromona gingivalis* strains were grown anaerobically (80% N_2_, 10% CO_2_, 10% H_2_) in enriched brain–heart infusion (BHI) broth (Becton Dickinson, Franklin Lakes, NJ) or on enriched tryptic soy (TS) agar plates (Nissui, Tokyo, Japan) supplemented with 5 μg/mL hemin (Sigma, St. Louis, MO) and 0.5 μg/mL menadione (Sigma). For blood agar plates, defibrinated laked sheep blood was added to enriched tryptic soy agar at 5%. Luria-Bertani (LB) broth and LB agar plates were used for growth of *Escherichia coli* strains. Antibiotics were used at the following concentrations: ampicillin (Ap; 100 μg/mL for *E. coli*, 10 μg/mL for *P. gingivalis*), erythromycin (Em; 10 μg/mL for *P. gingivalis*), gentamycin (Gm; 50 μg/mL for *P. gingivalis*), kanamycin (Km; 30 μg/mL for *E. coli*), spectinomycin (Sp; 80 μg/mL for *E. coli*), and tetracycline (Tc; 0.7 μg/mL for *P. gingivalis*).

### Chemicals

The proteinase inhibitors Nα-*p*-tosyl-l-lysine chloromethyl ketone (TLCK) and iodoacetamide were purchased from Wako (Japan), and leupeptin was obtained from the Peptide Institute (Japan).

### Sequence analysis

The genome sequence of *P. gingivalis* ATCC 33277 (GenBank: AP009380; Naito et al. 2008) was examined for the presence of the target gene with the transposon. Homology analysis was performed with BLAST. Secondary structure prediction of membrane proteins was performed using the SOSUI program (http://bp.nuap.nagoya-u.ac.jp/sosui/).

### Transposon mutagenesis

Tn*4400*' transposon mutagenesis of *P. gingivalis* ATCC 33277 was performed as described previously (Yamaguchi et al. 2010).

### Construction of *P. gingivalis* strains

The oligonucleotides used in this study are listed in Table S4. The general manipulation of DNA, restriction and mapping of plasmids, and transformation of *E. coli* and *P. gingivalis* were described in detail elsewhere (Shoji et al. 2010). The chromosomal DNA from *P. gingivalis* ATCC 33277 was used as the template for cloning purposes. The construction of various mutants from *P. gingivalis* ATCC 33277 or complemented strains from the mutants are described in Data S1 (Feldman et al. 1999; Nagano et al. 2007; Shi et al. 1999; Simon et al. 1983).

### Enzymatic assay

Kgp and Rgp activities were determined using the synthetic substrates benzyloxycarbonyl-l-histidyl-l-glutamyl-l-lysine-4-methyl-7-coumarylamide (Z-His-Glu-Lys-MCA) and carbobenzoxy-l-phenyl-l-arginine-4-methyl-7-coumarylamide (Z-Phe-Arg-MCA). The released 7-amino-4-methyl-coumarin was measured at 460 nm (excitation at 380 nm).

### Hemagglutination

Overnight cultures of *P. gingivalis* strains in enriched BHI medium were centrifuged, washed once with phosphate buffered saline (PBS), and suspended in PBS at an optical density of 0.5 at 595 nm. The bacterial suspensions were then diluted in a twofold series with PBS. A 100-μL aliquot of each suspension was mixed with an equal volume of defibrinated sheep erythrocyte suspension (1% in PBS) and incubated in a round-bottom microtiter plate at room temperature for 3 h.

### Gel electrophoresis and immunoblot analysis

SDS-PAGE and immunoblot analysis were performed as described previously (Shoji et al. 2010, 2011).

### Preparation of *P. gingivalis* LPS

Purification of the *P. gingivalis* LPS from the wild type, *porK*, *porT*, *porU*, *porV*, *porW*, *sov*, and *porR* mutants was performed by using an LPS purification reagent (Intron, Korea). As the amount of LPS from the PGN_2005 mutant was relatively small, a separate purification of LPS from the wild type or the PGN_2005 mutant was performed using the hot phenol method as described previously (Shoji et al. 2002). LPS was visualized by silver staining.

### Preparation of antiserum

Preparation of the anti-HBP35 used as anti-HBP35 rabbit polyclonal antibody (Abiko et al. 1990), mAb 1B5 used as anti-A-LPS (Curtis et al. 1999), and mAb TDC-5-2-1 used as anti-O-LPS (Maruyama et al. 2009) has been described previously. Preparation of mAb TDC-5-2-1 is described in detail in Data S1. To prepare the mouse antiserum against the peptides, one peptide corresponding to the amino acid region (E^361^-L^375^) within the catalytic domain of RgpB (PGN_1466), in which a cysteine residue was synthesized at the N-terminus of the peptide, was constructed and conjugated to keyhole limpet hemocyanin (Sigma Genosys, Tokyo, Japan). To raise antiserum against RgpB, mice were immunized by EveBioscience Co., Ltd. (Wakayama, Japan). To prepare mouse antiserum against the peptides corresponding to the C-terminal amino acid region of PGN_2005 (L^395^-Y^560^), the region of PGN_2005 was amplified with PGN_2005expFw/PGN_2005expBw and was cloned into the pET30 Ek/LIC vector (Novagen, Darmstadt, Germany), yielding pKD889. Then, pKD889 was transformed into *E. coli* BL21(DE3). The recombinant PGN_2005 (L^395^-Y^560^) protein expressed in *E. coli* BL21(DE3) was purified by His-tag affinity purification and injected into mice. The antiserum against PGN_2005 (L^395^-Y^560^) was collected from the immunized mice at Biomedical Research Centre, Centre for Frontier Life Sciences in Nagasaki University. Animal care and experimental procedures were performed in accordance with the Guidelines for Animal Experimentation of Nagasaki University with approval from the Institutional Animal Care and Use Committee.

### Cell fractionation analysis

Sample preparation and all procedures were followed as described previously (Murakami et al. 2002).

### Sucrose density gradient centrifugation

A whole cell envelope suspension (1.5 mL) from the wild-type fully grown culture (200 mL) was applied to the discontinuous sucrose density gradient (1.6 mL of 2.02 mol/L, 5.6 mL of 1.44 mol/L, and 4.0 mL of 0.77 mol/L sucrose in HEPES buffer) as described previously (Murakami et al. 2002). The gradients were centrifuged at 100,000*g* for 48 h at 4°C in a Beckman Coulter (Brea, CA) SW41 rotor. Fractions (0.5 mL) were collected from the top of the centrifuge tube. NADH-dependent ferricyanide reductase activity was measured as an indicator for the inner membrane by the method described by Futai (1974).

### In vivo complementation analysis

Strain EVV16 (*Escherichia coli* W3110 Δ*wzzB*::Km [Vinés et al. 2005]) was used for in vivo complementation analysis. Bacteria were cultured at 37°C in LB medium supplemented with Ap (100 μg/mL), Km (30 μg/mL), Sp (80 μg/mL), and 0.2% (wt/vol) arabinose as appropriate. The cloning of the PGN_2005 gene was performed by amplifying its coding region using the forward primer (5′-CCATGGCCATGACTGAGAAATCATTTCGAA-3′) carrying a NcoI site (underlined) located 8 bases upstream of the PGN_2005 start codon (double underlined) and the reverse primer (5′-AAGCTTTTAATACCTATCCAACCATAGCAC-3′), which included a HindIII site (underlined) and a stop codon (double underlined). The PCR product was cloned into pUC118, yielding pKD890. The PGN_2005 gene region was digested with NcoI and HindIII from the pKD890 and was cloned into the same sites of pBAD/Myc-His A (Invitrogen, Carlsbad, CA), yielding pKD891. pMF19-containing rhamnosyltransferase was introduced into *E. coli* W3110 and EVV16. Then, each complementation plasmid was introduced into *E. coli* cells. *Escherichia coli* LPSs were extracted using an LPS purification reagent (Intron, Korea). LPS was resolved by electrophoresis in a 10% SDS-polyacrylamide gel and visualized by silver staining.

### Phylogenetic tree analysis of the Wzz proteins

The phylogenetic tree of the Wzz proteins was constructed by using the concatenated or whole amino acid sequences of Wzz from various bacterial strains. The amino acid sequences of the Wzz family were aligned, and ambiguous portions of the alignment were removed using BioEdit Sequence Alignment Editor software. MEGA5.05 software was used to generate the phylogenetic tree using the neighbor-joining method.

## Results

### Identification of a gene disrupted by Tn*4400*' transposon insertion

**Figure 1:**
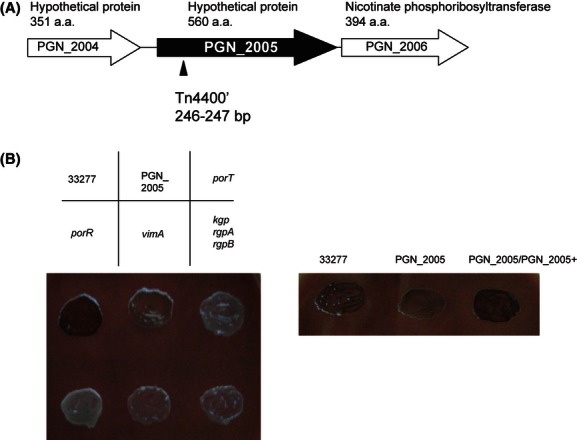
Physical map of the area around the PGN_2005 gene and pigmentation of PGN_2005 mutant. Physical map of the PGN_2005 gene region (A). A triangle indicates the Tn*4400*' insertion site of the PGN_2005 insertion mutant. Colony pigmentation (B). *Porphyromonas gingivalis* cells were anaerobically grown on blood agar plates at 35°C for 2 days.

A collection of *P. gingivalis* ATCC 33277 random-transposon mutants was screened on blood agar plates for colonial pigmentation. The chromosomal DNA of a less-pigmented mutant (KDP205) was digested with HindIII, self-ligated and cloned into *E. coli* by the marker rescue method with selection for the *bla* gene in Tn*4400*' DNA. The sequencing of the cloned DNA fragment revealed that the insertion site of the transposon was located 246 bp downstream from the first nucleotide residue of the initiation codon of PGN_2005 (Fig. 1A). Complementation analysis revealed that the complemented strain was more pigmented than the PGN_2005 mutant (Fig. 1B).

**Figure 2:**
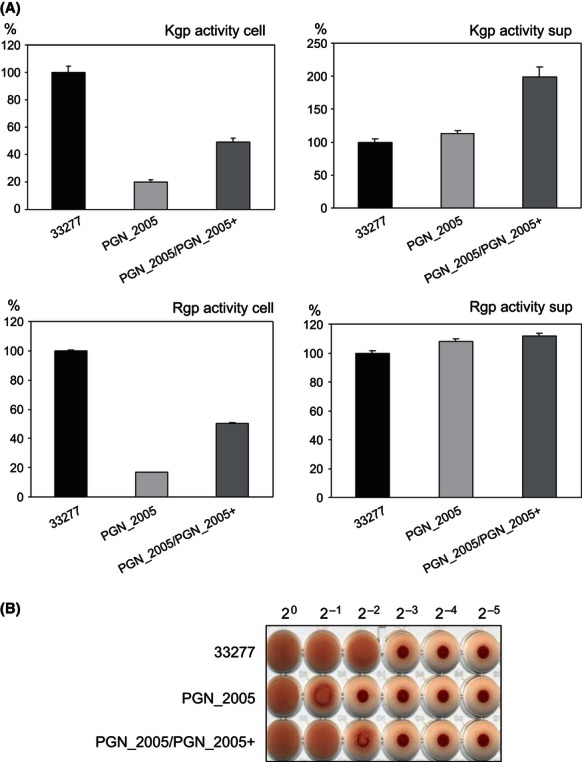
Gingipain and hemagglutination activities of *Porphyromonas gingivalis*. *Porphyromonas gingivalis* cells were anaerobically grown in enriched BHI medium at 35°C. The Kgp and Rgp activities of the cell lysates (cell) and vesicle-containing culture supernatants (sup) of ATCC 33277 (wild type), PGN_2005, or PGN_2005/PGN2005+ were measured (A). The hemagglutination activities of various *P. gingivalis* strains were measured (B). Twofold serial dilutions of various *P. gingivalis* cells were mixed with 1% sheep red blood cells and stored for 3 h at room temperature.

We next examined Rgp and Kgp protease activities (Fig. 2A). The cell-associated Rgp and Kgp activities of the PGN_2005 mutant decreased by nearly 80% compared with that of the wild type. In the complemented strain, the partial recovery of the cell-associated gingipain activities relative to the PGN_2005 mutant was achieved. We did not observe significant differences in the Rgp and Kgp activities from the culture supernatants between the wild type and the PGN_2005 mutant (Fig. 2A). The Kgp activity from the culture supernatant of the complemented strain was twofold more than that of the wild type (Fig. 2A). In addition, we examined the hemagglutination activity of the *P. gingivalis* strains (Fig. 2B). The hemagglutination titer of the PGN_2005 mutant was twofold less than that of the wild type.

The PGN_2005 gene product has been annotated as a hypothetical protein of *P. gingivalis* ATCC 33277 (Naito et al. 2008). Homology searches revealed that genes orthologous to PGN_2005 are present in *Porphyromonas asaccharolytica* (Poras_0604 in strain DSM20707), *Porphyromonas uenosis* (PORUE0001_0693 in strain 60-3), and *Porphyromonas endodontalis* (POREN0001_1785 in strain ATCC 35406; Fig. S1). An analysis with the SOSUI program predicted that the PGN_2005 protein is located in the inner membrane and has two transmembrane regions (Fig. S1).

### Profiles of two LPSs of the PGN_2005 mutant display lower bands than those of the wild type

**Figure 3:**
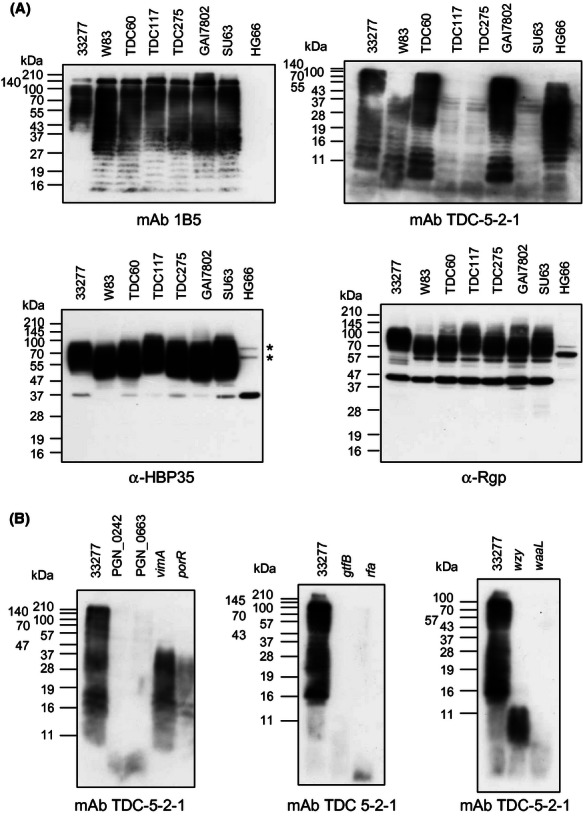
Immunoblot analyses of various *Porphyromonas gingivalis* strains. The cell lysates of various *P. gingivalis* strains were subjected to SDS-PAGE, and immunoblot analyses were performed with anti-HBP35, anti-Rgp, mAb1B5, or mAb TDC-5-2-1. The asterisks indicate nonspecific cross-reactive bands (A). Immunoblot analyses of various *P. gingivalis* A-LPS-deficient mutants. Cell lysates of various *P. gingivalis* A-LPS-deficient mutants were subjected to SDS-PAGE, and immunoblot analysis was performed with mAb TDC-5-2-1 (B).

**Figure 4:**
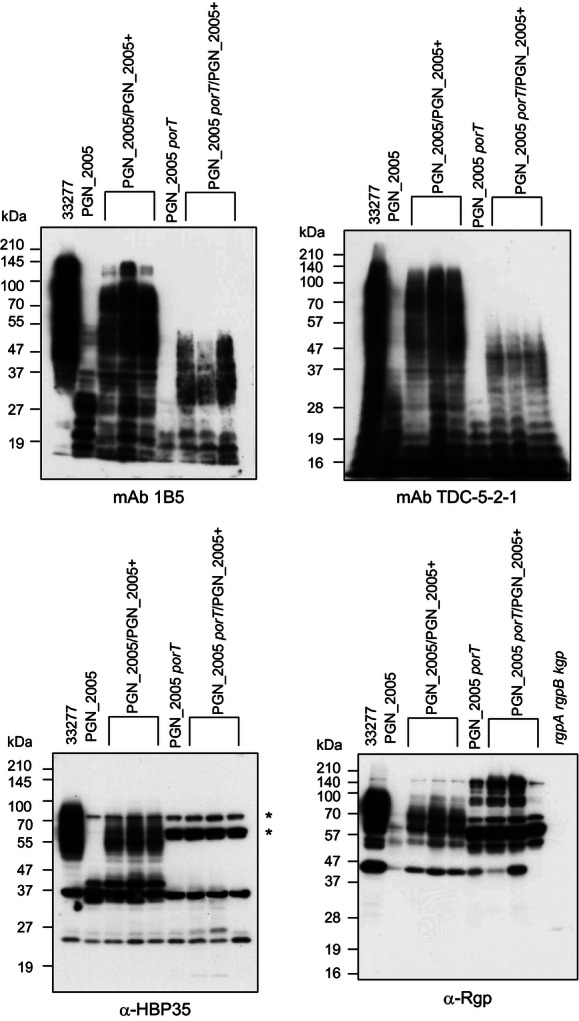
Immunoblot analyses of various *Porphyromonas gingivalis* strains. Immunoblot analyses of cell lysates of various *P. gingivalis* strains were performed with mAb 1B5, mAb TDC-5-2-1, anti-HBP35, or anti-Rgp. Three sets of PGN_2005/PGN_2005+ or PGN_2005 porT/PGN_2005+ strains were obtained from each single clone. The asterisks indicate nonspecific cross-reactive bands.

**Figure 5:**
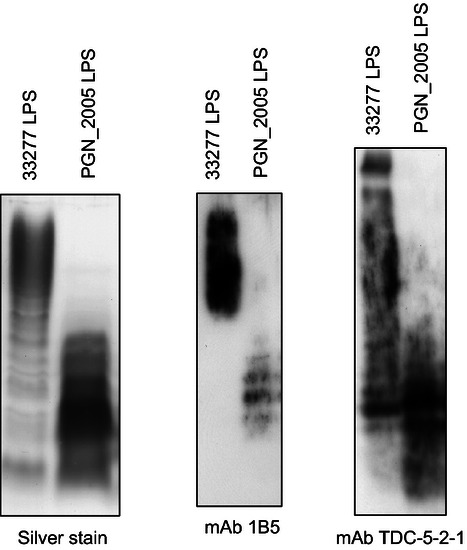
Purified LPS from the wild type or the PGN_2005 mutant. The LPS fraction was purified by the hot phenol method and subjected to SDS-PAGE followed by silver staining. Immunoblot analyses were also performed with mAb 1B5 and mAb TDC-5-2-1.

The decrease of cell-associated Rgp and Kgp activities and the lack of decrease of secreted Rgp or Kgp activity in the PGN_2005 mutant suggested that the PGN_2005 mutant should be categorized as a *porR*-type mutant. We then examined the PGN_2005 mutant for LPS. MAb 1B5, which recognizes a glycan epitope of anionic polysaccharide bound to lipid A (called A-LPS), has been successfully used in the past as an anti-A-LPS antibody. In addition to mAb 1B5, we used mAb TDC-5-2-1 in this study, which recognizes LPS containing a normal polysaccharide O-side chain bound to lipid A (now referred as O-LPS). This was indicated by the following results: (i) mAb TDC-5-2-1, which was obtained by inoculation of cell extracts from *P. gingivalis* TDC60 to mice, reacted to LPS purified from the strain with a ladder-like structure pattern (Maruyama et al. 2009); (ii) mAb TDC-5-2-1 strongly reacted to cell lysates of *P. gingivalis* ATCC 33277, W83, TDC60, GAI7802, and HG66 with a ladder-like structure pattern and weakly reacted to lysates of strains TDC117, TDC275, and SU63 (Fig. 3A); (iii) cell lysates of *P. gingivalis* strain HG66, which displayed no reactivity against mAb 1B5, were recognized by mAb TDC-5-2-1 (Fig. 3A); and (iv) mAb TDC-5-2-1 recognized the low-molecular-mass product(s) of the cell lysates of the PGN_1242 (*wzy*) mutant but not those of the PGN_1251 (*gtfB*) and PGN_1302 (*waaL*) mutants (Fig. 3B). LPS from the *wzy* mutant possesses one repeat unit of O-antigen attached to a lipid A-core, whereas LPSs from the *waaL* and *gtfB* mutants possess a lipid A-core only and a truncated one-repeat unit of O-antigen attached to a lipid A-core, respectively. As the difference between the *wzy* and *waaL* mutants is one repeat unit of O-antigen only, mAb TDC-5-2-1 appears to recognize a glycan epitope within one repeat unit of O-antigen in O-LPS. To determine whether the PGN_2005 mutant has any defects in LPS biosynthesis or glycosylated CTD proteins, such as HBP35 and RgpB, we performed immunoblot analyses with mAb 1B5, mAb TDC-5-2-1, anti-HBP35, and anti-Rgp antibodies, respectively (Fig. 4). Both mAb 1B5 and mAb TDC-5-2-1 recognized low-molecular-mass products in the PGN_2005 mutant cell lysate compared with the wild type. We have recently shown that the HBP35 is secreted via the PorSS and is glycosylated with A-LPS, which is observed as diffuse bands on an SDS-PAGE gel (Shoji et al. 2010, 2011). Therefore, we examined whether the HBP35 in the PGN_2005 mutant is glycosylated. Only discrete 41-, 40-, and faint 39-kDa HBP35 bands were observed, but not the diffuse bands of HBP35 in the PGN_2005 mutant (Fig. 4). The diffuse bands of RgpB were also not present in the PGN_2005 mutant, as revealed by immunoblot analysis with anti-Rgp antibody (Fig. 4). The complemented strain displayed similar immunoreactive bands when mAb 1B5 or mAb TDC-5-2-1 was used to test against the wild-type cell lysate (Fig. 4). Furthermore, we also confirmed that LPS purified from the PGN_2005 mutant by a hot phenol method is shorter than that of the wild type and forms less than 10 repeat units of O-antigen linked to lipid A-cores (Fig. 5). With these results taken into consideration, the PGN_2005 protein is likely to be a homolog of Wzz protein, which belongs to the polysaccharide copolymerase family. These proteins play a role in determining the chain length of O-antigen.

### The PGN_2005 protein localizes in the inner membrane

**Figure 6:**
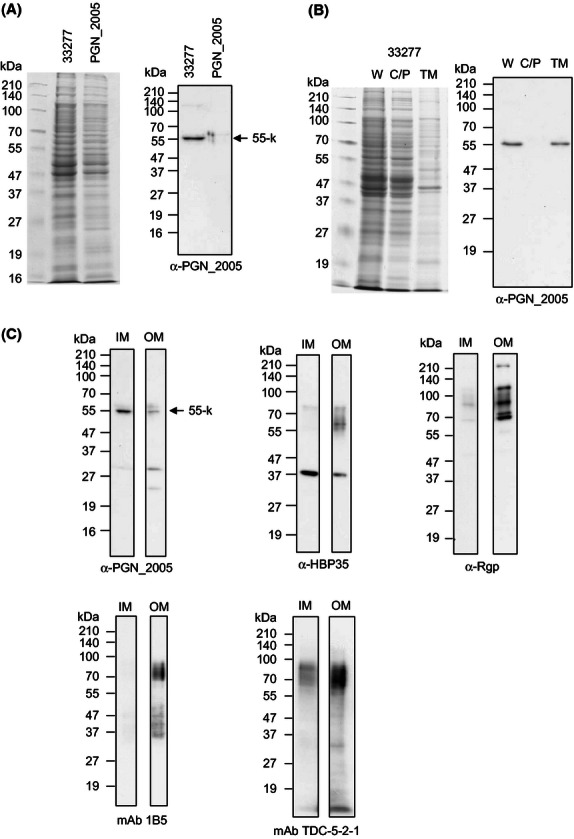
Localization of the PGN_2005 protein in *Porphyromonas gingivalis*. The cell lysates of the wild type and the PGN_2005 mutant were subjected to immunodetection with anti-PGN_2005 (A). Cell fractionation analysis from the wild type. W, C/P, and TM indicate the whole cell lysate, cytoplasm/periplasm, and total membrane fraction, respectively (B). Five micrograms of protein from the inner membrane (IM) or outer membrane (OM) fractions that were separated by sucrose density gradient centrifugation from the membrane fraction of the wild type were subjected to immunodetection with anti-PGN_2005, anti-HBP35, anti-Rgp, mAb 1B5, and mAb TDC-5-2-1 (C).

As the PGN_2005 protein is predicted to possess two transmembrane regions but not a typical signal sequence in the N-terminus as the Wzz proteins do and to localize in the inner membrane, we examined whether the PGN_2005 protein localized in the inner membrane. The C-terminal cytoplasmic region of the PGN_2005 protein is longer than that of other Wzz proteins. We obtained an anti-PGN_2005 antibody against the C-terminal cytoplasmic region of the PGN_2005 protein. Immunoblot analysis revealed that the anti-PGN_2005 antibody recognized the 55-kDa protein in the wild type, but not in the PGN_2005 mutant (Fig. 6A). Although the apparent molecular mass of 55 kDa for the monomer is considerably lower than the predicted value of 63.2 kDa, such aberrations in molecular masses have frequently been encountered in SDS-PAGE analyses of integral membrane proteins (Abeyrathne and Lam 2007). Cell fractionation analysis revealed that the 55-kDa protein was observed in the total membrane fraction (Fig. 6B). Next, we separated the total membrane fraction of the wild type into inner and outer membrane fractions by sucrose density gradient centrifugation. We collected 20 fractions starting from the top of the centrifuge tube. Then, we measured the NADH-dependent ferricyanide reductase activity, which is characteristic of the inner membrane fraction. Fraction number 9, which displayed the highest NADH-dependent ferricyanide reductase activity, was used as the inner membrane fraction, and fraction number 20 was used as the outer membrane fraction (justified below). As shown in Figure 6C, the 55-kDa PGN_2005 protein of the inner membrane fraction was observed more than that of the outer membrane fraction. As we reported previously (Shoji et al. 2010), the outer membrane fraction, as separated by sucrose density gradient centrifugation, contained diffuse bands of HBP35 and RgpB and also displayed the presence of A-LPS and O-LPS. These results indicated that the PGN_2005 protein was located in the inner membrane, which is consistent with the localization of Wzz proteins.

### PGN_2005 protein confers Wzz activity upon an *E. coli wzz* mutant

**Figure 7:**
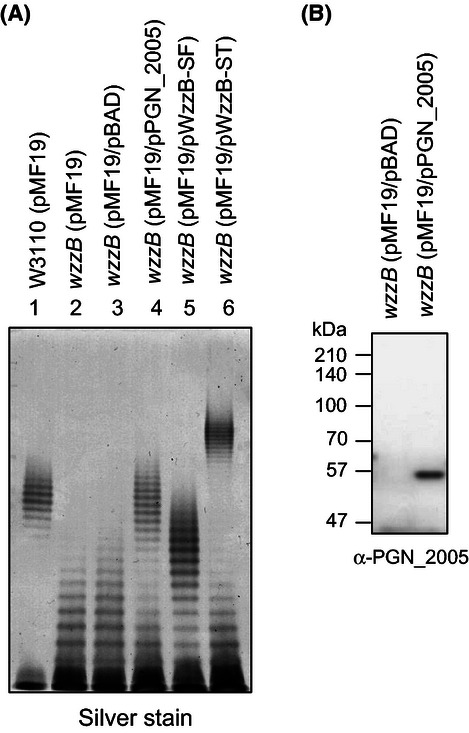
Analysis of LPS to assess Wzz activity by heterologous complementation. Silver-stained polyacrylamide gel displaying the O-antigen LPS profiles of *Escherichia coli* W3110 (lane 1), EVV16 (*wzzB*) containing pMF19 (lane 2), EVV16/pMF19 containing pBAD vector control (lane 3), EVV16/pMF19 containing PGN_2005-expressing plasmid from *Porphyromonas gingivalis* (lane 4), EVV16/pMF19 containing WzzB-expressing plasmid (pWzzB-SF) from *Shigella flexneri* (lane 5), and EVV16/pMF19 containing WzzB-expressing plasmid (pWzzB-ST) from *Salmonella typhimurium* (lane 6) (A). Immunoblot analysis of the cell lysates was performed with anti-PGN_2005 mouse polyclonal antiserum to confirm the expression of the PGN_2005 protein in the *E. coli* EVV16 (pMF19) strain (B).

To determine whether the PGN_2005 protein has Wzz activity, we conducted a heterologous complementation analysis using an *E. coli wzzB* mutant. As shown in Figure 7, the PGN_2005 protein, which is expressed in *E. coli*, plays a role in determining the chain length of the *E. coli* O-antigen. The Wzz activity of PGN_2005 is between that of WzzB-ST, which is from *Salmonella typhimurium*, and that of WzzB-SF, which is from *Shigella flexneri*. We designated the PGN_2005 protein WzzP.

### Comparison of the amino acid sequence of *P. gingivalis* WzzP with those of Wzz proteins of other bacteria

**Figure 8:**
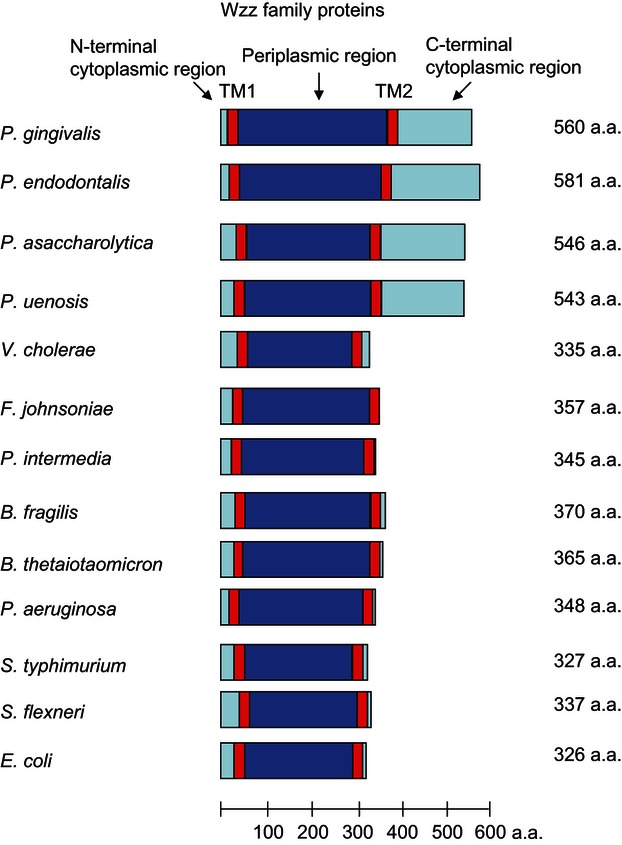
Comparison of Wzz family proteins. The transmembrane regions of various Wzz family proteins were analyzed using the SOSUI program.

Wzz proteins of other bacteria such as *E. coli* and *Bacteroides fragilis* consist of 300–400 amino acids, whereas *P. gingivalis* WzzP consists of 560 amino acids (Fig. 8). A comparison of the complete amino acid sequences of those Wzz proteins revealed that WzzP shared weak similarity with the Wzz proteins in other bacteria and that the Wzz protein had a long C-terminal cytoplasmic region consisting of approximately 160 amino acid residues, while other Wzz proteins contained short C-terminal cytoplasmic regions consisting of approximately 10 amino acid residues. These differences were conserved among WzzP homologs in the genus *Porphyromonas*. The C-terminal cytoplasmic region of WzzP has no sequence similarity to any proteins other than the WzzP-like proteins. We then compared these proteins in the forms lacking the nonhomologous C-terminal regions. The results were similar to that of the whole length comparison (Fig. S2).

### WbaP and Wzx proteins are involved in A-LPS biosynthesis

**Figure 9:**
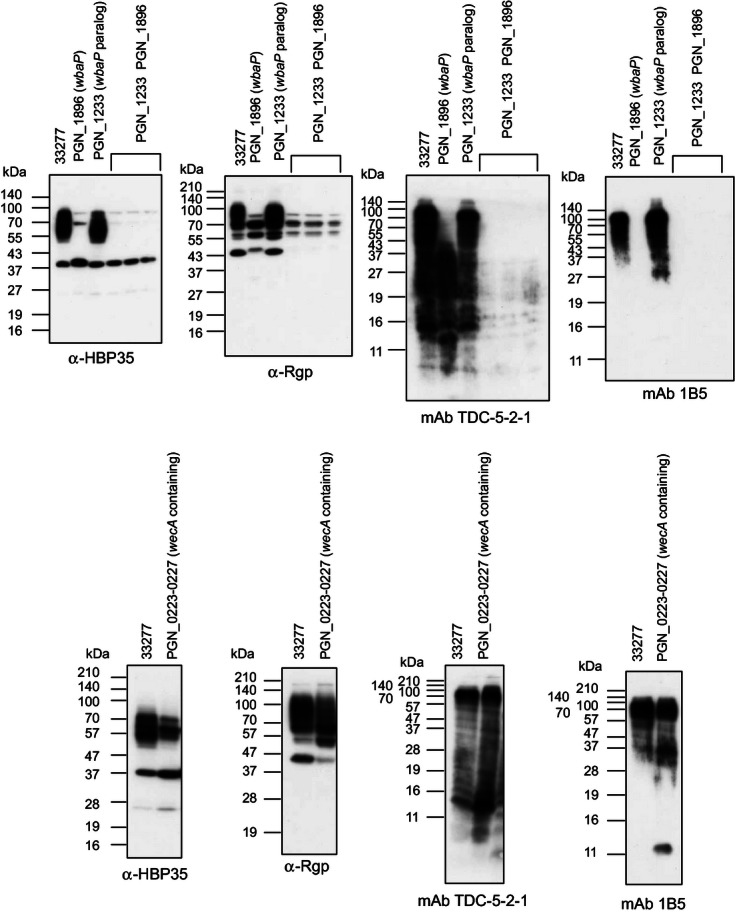
Immunoblot analyses of various *Porphyromonas gingivalis* mutants related to the first initiation enzyme of UndPP-glycan with anti-HBP35, anti-Rgp, mAb 1B5, and mAb TDC-5-2-1. Cell lysates of various *P. gingivalis* mutants were subjected to SDS-PAGE and immunoblot analysis with anti-HBP35, anti-Rgp, mAb 1B5, and mAb TDC-5-2-1.

A homology search revealed that PGN_1233 and PGN_1896 were putative WbaP homolog proteins and that PGN_0223 was a putative WecA homolog protein. To determine which genes were involved in the biosynthesis of the two LPSs, we constructed the PGN_1233 (putative *wbaP* paralog), PGN_1896 (putative *wbaP*), and PGN_0223-0227 (putative *wecA*- UDP-N-acetyl-d-mannosaminuronic acid dehydrogenase-glycosyl transferase-hypothetical protein-glycosyl transferase) mutants. As shown in Figure 9, the PGN_1896 mutant presented no immunoreaction to mAb 1B5 and no diffuse bands of HBP35 and RgpB. Interestingly, the molecular masses of the immunoreactive products of the PGN_1896 mutant probed with mAb TDC-5-2-1 decreased, similar to the phenotype of the *porR* mutant. This result suggests that the PGN_1896 gene product contributes to the biosynthesis of the glycan epitope of mAb 1B5. The immunoreactive products of the PGN_1233 mutant were similar to those of the wild type. To reveal the significances of the PGN_1233 and PGN_1896 genes, we constructed a PGN_1233 PGN_1896 double mutant. The immunoreactive products of the PGN_1233 PGN_1896 double mutant probed with mAb TDC-5-2-1 were significantly less than was observed with PGN_1233 and PGN_1896 single mutants, suggesting that both the PGN_1233 and PGN_1896 genes play a role in the biosynthesis of the glycan epitope of mAb TDC-5-2-1. However, the phenotype of the PGN_0223-0227 deletion mutant including the PGN_0223 region was similar to that of the wild type, suggesting that the PGN_0223 gene product does not play a role in the biosynthesis of the glycan epitopes of mAbs 1B5 and TDC-5-2-1, respectively.

**Figure 10:**
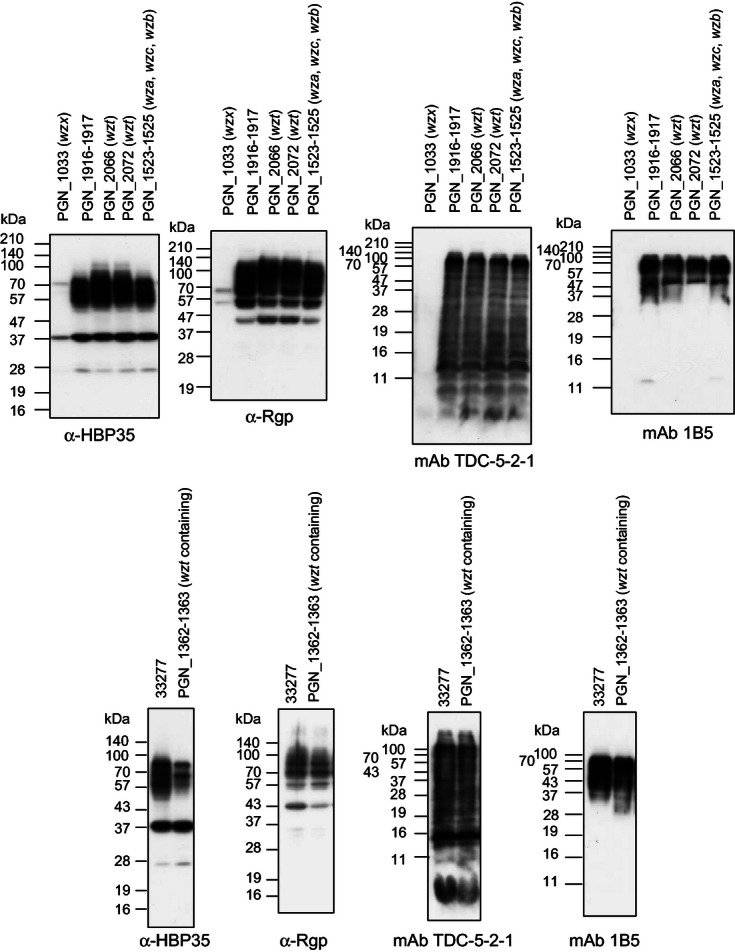
Immunoblot analysis of various *Porphyromonas gingivalis* mutants related to the O-antigen flippase with anti-HBP35, anti-Rgp, mAb 1B5, and mAb TDC-5-2-1. Cell lysates of various *P. gingivalis* mutants were subjected to SDS-PAGE and immunoblot analysis with anti-HBP35, anti-Rgp, mAb 1B5, and mAb TDC-5-2-1.

As PGN_1242 and PGN_1302 have been identified as Wzy and WaaL, respectively, and PGN_2005 was identified as a Wzz protein in this study, we focused on PGN_1033 (putative *wzx*), PGN_1917-1916 (putative ABC transporter–ABC transporter), PGN_2066 (putative *wzt*), PGN_2072 (putative *wzt*), PGN_1523-1525 (putative *wza-wzc-wzb*), and PGN_1362-1363 (putative exported transglycosylase protein-*wzt*) genes and constructed the relevant mutants to determine which genes were involved in LPS biosynthesis. We found that neither of the monoclonal antibodies recognized any products of the PGN_1033 mutant (Fig. 10). The diffuse bands of HBP35 and RgpB were also not observed with the PGN_1033 mutant (Fig. 10). These results suggest that the PGN_1033 gene product plays a part in the biosynthesis of the two LPSs and belongs to the Wzx protein family, and acts as an UndPP-glycans flippase, although we have no experimental evidence of the enzymatic activity.

## Discussion

LPS is composed of O-antigen, core polysaccharide, and lipid A. Biosynthesis of the O-antigen in *P. gingivalis* is assumed to occur by Wzy- and WaaL-dependent pathway (Paramonov et al. 2009). Briefly, the initiating reaction to form UndPP-glycan occurs via WbaP or WecA, which functions as a galactose-1-phosphate transferase and a GlcNAc-1-phosphate transferase, respectively. Then, UndPP-glycans are achieved on the cytoplasmic side of the inner membrane by specific glycosyltransferases, and are transported onto the periplasmic side of the inner membrane via a Wzx flippase located in the inner membrane. Polymerization of O-antigen occurs in the periplasm and is dependent on the inner membrane polymerase Wzy. The resulting O-antigen chains are typically clustered around a certain number of O-antigen repeat units determined by Wzz, the chain length regulator, also located in the inner membrane (Hug and Feldman 2011). After polymerization, the O-antigen chains are ligated to the preformed lipid A-core by the inner membrane ligase, WaaL, and the mature LPS is transported to the outer membrane by LPS transport proteins (Sperandeo et al. 2009). On the other hand, it has been shown that polymeric O-antigen assembly can also occur by an ATP-binding cassette (ABC) transporter-dependent pathway. Generally, the ABC transporter formed by Wzm and Wzt, which are the permease and ABC transporter, respectively, or a single protein, such as Wzk, is required for the transfer of the undecaprenyl-linked polymer to the periplasmic side of the inner membrane, where it is ligated to the lipid A-core and translocated to the outer membrane (Hug and Feldman 2011).

In this study, we isolated a novel pigment-less mutant by transposon mutagenesis. The transposon-inserted gene was identified as the PGN_2005 gene, the product of which is annotated as a hypothetical protein of *P. gingivalis* ATCC 33277 (Naito et al. 2008). The PGN_2005 protein appears to be a functional homolog of Wzz proteins for the following reasons: (i) the PGN_2005 protein was located in the inner membrane (Fig. 6); (ii) the secondary structure prediction of the amino acid sequence of PGN_2005 indicated that the protein has two transmembrane regions and one extended periplasmic loop region (Figs. 8 and S1); (iii) the PGN_2005 protein shared weak amino acid sequence homology with conventional Wzz proteins of other bacteria (Fig. S2); (iv) cell lysate products or purified LPS from the PGN_2005 mutant reacted with the LPS-specific antibodies mAb 1B5 and mAb TDC-5-2-1, resulting in shorter bands than those observed in the wild type (Figs. 4 and 5); and (v) the PGN_2005 gene conferred Wzz activity upon an *E. coli wzz* mutant (Fig. 7). We designated the PGN_2005 gene *wzzP*. The WzzP-like proteins can be found in *P. asaccharolytica* (Poras_0604), *P. uenosis* (PORUE0001_0693), and *P. endodontalis* (POREN0001_1785). Interestingly, all the bacteria belong to the genus *Porphyromonas*. The flanking genes of the *wzzP* homologs are also identical among *P. gingivalis*, *P. asaccharolytica*, *P. endodontalis*, and *P. uenosis*; however, they are unlikely to be involved in LPS biosynthesis. Most of the genes involved in LPS biosynthesis are scattered in the chromosomes of bacteria belonging to the phylum *Bacteroidetes* (Table S1).

It has been shown that Wzz proteins determine the chain length of O-antigen polysaccharides synthesized by Wzy. In the absence of Wzz, modality, that is, the nonrandom chain length of O-antigen, is lost, and predominantly short or long polysaccharides are produced (Bastin et al. 1993; Morona et al. 1995). A successive in vitro bacterial polysaccharide biosynthesis assay using Wzy and Wzz proteins has been reported (Woodward et al. 2010). And very recently, Kalynych et al. (2012) proposed that Wzz proteins bind and stabilize the growing O-antigen intermediates and prevent the premature release of the growing O-antigen, and once the O-antigen contains the appropriate number of the repeat units that can no longer be bound by Wzz, it is released. Most of Wzz proteins are 36- to 40-kDa inner membrane proteins with variable sequence identity (approximately 15–80%), whereas the WzzP protein of *P. gingivalis* consists of 560 amino acids and produces a 55-kDa protein on an SDS-PAGE gel (Figs. 6 and 7). The main difference between the WzzP protein and Wzz proteins is that the WzzP protein has a long C-terminal cytoplasmic region consisting of approximately 160 amino acid residues, while Wzz proteins contain a short C-terminal cytoplasmic region consisting of approximately 10 amino acid residues (Fig. 8). BLAST searches revealed that the C-terminal cytoplasmic region of WzzP has no sequence similarity to any proteins other than the WzzP-like proteins. The C-terminal cytoplasmic region of the WzzP protein may not play an important role in Wzz activity. It has been shown that the oligomerization state of Wzz proteins is critical for their function (Tang et al. 2007; Papadopoulos and Morona 2010) and that several periplasmic regions of the proteins are important for the activity (Kalynych et al. 2011). The WzzP protein may form the oligomerization state and function with Wzy; however, the periplasmic loop region of WzzP has a weak sequence similarity to those of Wzz proteins.

It has been reported that the Wzz proteins in *Vibrio cholera* and *B. fragilis* play roles in the biosynthetic pathways of the O-antigen capsule and the high-molecular-mass polysaccharides, respectively (Attridge et al. 2001; Patrick et al. 2009). To determine the effect of *P. gingivalis* WzzP on capsular polysaccharides, we constructed a *wzzP* (PG0056) mutant from strain W83 possessing K1, the capsular antigen, as strain ATCC 33277 lacks the K1 antigen. Similar to the ATCC 33277 *wzzP* mutant, the W83 *wzzP* mutant presented shorter bands that were immunoreactive to mAbs 1B5 and TDC-5-2-1. The W83 *wzzP* mutant was shown to be encapsulated (data not shown), suggesting that WzzP plays a role in the biosynthesis of LPS, but not in that of capsular polysaccharide. Wzc homolog protein (PGN_1524) has weak similarity to WzzP. Wzc proteins possess two transmembrane regions and a long C-terminal cytoplasmic domain and undergo the transphosphorylation of several C-terminal tyrosine residues to translocate the capsular polysaccharides (Whitfield 2006). In contrast, WzzP proteins do not possess such a tyrosine-rich region. Several bacteria, such as *S. typhimurium*, *S. flexneri*, and *Pseudomonas aeruginosa*, possess two types of Wzz proteins, and the Wzz proteins differentially regulate the length of LPS (Morona et al. 2003; Murray et al. 2003; Kintz et al. 2008). In *P. gingivalis*, no *wzzP* paralogs were identified in the genome. LPSs with various O-antigen chain lengths produced by Wzz proteins are important for resistance to complement (Murray et al. 2006). WzzP may be crucial for the resistance of *P. gingivalis* to complement, as PorR is responsible for complement resistance (Slaney et al. 2006).

**Figure 11:**
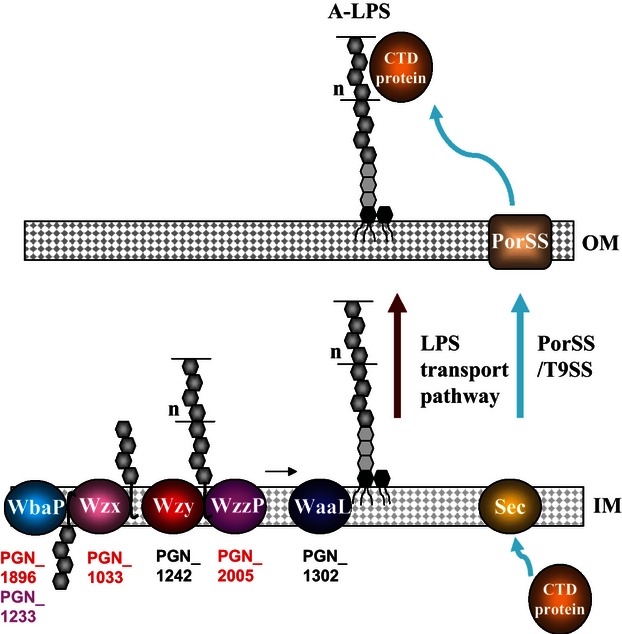
Transport model of LPS and CTD proteins. The first initiation enzymes of UndPP-glycan for two LPSs in *Porphyromonas gingivalis* are WbaP-like proteins (PGN_1896 and PGN_1233). Assembly of UndPP-glycans is achieved at cytoplasmic side of the inner membrane, and the block is then transported onto the periplasmic side of the inner membrane by Wzx (PGN_1033). The nonrandom (modal) chain length of O-antigen is dictated by Wzy and Wzz proteins, which correspond to an O-antigen polymerase (PGN_1242) and O-antigen chain length regulator (PGN_2005), respectively. Then, O-antigen is ligated to preformed lipid A-cores by O-antigen ligase (PGN_1302), resulting in LPS. LPS is transported to the outer membrane by LPS transport proteins, which are poorly characterized in *P. gingivalis*. The C-terminal domain proteins are transported to the outer membrane by Sec and the Por secretion system/Type IX secretion system (PorSS/T9SS). Currently, the precise glycosylation mechanism of the CTD proteins remains uncertain.

Nakao et al. (2006) constructed *wbaP* homolog (PGN_1896), *wecA* homolog (PGN_0223), and *wzt* homolog (PGN_1363) mutants from strain ATCC 33277 by insertion of the *ermF*-*ermAM* cassette into the target genes and found that the distribution of the O-antigen ladder in the mutants did not change compared with that of the wild type. We have sometimes found that the distribution of the O-antigen ladder is different between an insertion mutant and a deletion mutant; this difference is presumably due to residual activity of the insertion mutant. To exclude this residual activity, we constructed deletion mutants of PGN_1896 (*wbaP* homolog), PGN_1233 (*wbaP* homolog), PGN_1033 (*wzx* homolog), PGN_1362-1363 (exported transglycosylase-*wzt* homolog), PGN_2066 (*wzt* homolog), PGN_2072 (*wzt* homolog), PGN_1916-1917 (putative ABC transporter–putative ABC transporter), and PGN_1523-1525 (*wza*-*wzc*-*wzb* homologs encoding capsular polysaccharide transporter). We found that WbaP homolog (PGN_1896) is critical for A-LPS biosynthesis, and Wzx homolog (PGN_1033) is critical for both biosynthesis of A-LPS and O-LPS (Figs. 1). As mAb TDC-5-2-1-immunoreactive products significantly decrease in PGN_1896 and PGN_1233 (*wbaP* homologs) double-mutant cell lysates, both PGN_1896 and PGN_1233 genes are important for the formation of the glycan epitope recognized by mAb TDC-5-2-1. However, the double mutant had faint ladder bands with mAb TDC-5-2-1, suggesting that other initiating enzyme(s) that can form UndPP-glycan are present (Fig. 9). It has recently been shown that PorS (PGN_1235) possesses Wzx flippase activity (Haurat et al. 2011). As cell lysates of the *porS* mutant were still immunoreactive with mAb 1B5, albeit at a much reduced level, other gene(s) responsible for the Wzx activity may exist. We found that PGN_1033 is critical for the biosynthesis of both A-LPS and O-LPS, suggesting that it acts as an O-antigen flippase, as it has high similarity to other Wzx proteins (Fig. 11).

Recently, it has been shown that mature LPS is transported to the outer membrane by the “Lpt machinery.” LPS transport proteins involved in the process are located at the inner membrane (LptB, LptC, LptF, and LptG), in the periplasm (LptA), and at the outer membrane (LptD and LptE) (Sperandeo et al. 2009; Chng et al. 2012; Okuda et al. 2012). We found that PGN_1553 contains LptA-, LptC-, or LptD-like domains, PGN_0669, PGN_1512, PGN_0884, and PGN_0260 are the best-matched *P. gingivalis* equivalents of LptB, LptC, LptD, and LptE, respectively, and PGN_0642 contains LptF- or LptG-like domains. Genes encoding Lpt proteins are essential in *E. coli*, but not in *Neisseria meningitides* (Bos and Tommassen 2011). As we have failed to construct deletion mutants for PGN_1553 and PGN_0884 thus far, it is possible that these genes may encode essential Lpt proteins (data not shown).

*Porphyromonas gingivalis* displays black pigmentation on blood agar plates. The black pigmentation is the result of storage of the μ-oxo-dimeric form of heme (iron protoporphyrin IX) on the cell surface (Smalley et al. 1998). It has been shown that Lys-gingipain can degrade hemoglobin protein, which holds heme molecules; this correlates with the inability to pigment, as a mutant with the *kgp* gene encoding a Lys-gingipain displayed a pigment-less phenotype on blood agar plates (Okamoto et al. 1998; Curtis et al. 2002). Our previous study demonstrated that the PorT protein is critical for the secretion of the gingipain-related proteins, which consist of the Arg-gingipains, Lys-gingipain, and HagA (Sato et al. 2005). These proteins have a conserved CTD in their primary sequences (Seers et al. 2006). Via genome analysis, we revealed that *P. gingivalis* strains have 34 and 33 proteins, including a conserved C-terminal domain, in strains W83 and strain ATCC 33277, respectively. For the CTD family proteins, the CTD region is required for proper secretion and attachment onto the cell surface. We recently have shown that *P. gingivalis* has a novel secretion apparatus, termed the PorSS (T9SS) (Sato et al. 2010). This system consists of 11 proteins, including PorK, PorL, PorM, PorN, PorP, PorQ, PorT, PorU (Glew et al. 2012), PorV/Pg27/LptO (Ishiguro et al. 2009; Chen et al. 2011), PorW, and Sov (Saiki and Konishi 2007), and plays a role in secreting the CTD proteins. It has been shown that RgpB, TapA, HBP35, and CPG70, which belong to the CTD family proteins, present diffuse bands on SDS-PAGE gels (Nguyen et al. 2007; Kondo et al. 2010; Shoji et al. 2010; Chen et al. 2011). Through GFP-fusion analysis, we recently demonstrated that 22 C-terminal amino acids of HBP35 are required to secrete via the PorSS (T9SS) and form the diffuse bands in the wild type (Shoji et al. 2011). In the PGN_2005 mutant, we found that Arg-gingipain and the HBP35 protein are secreted via the PorSS (T9SS), and relatively small amounts of the proteins are associated with the cell (Fig. 4). *porR* and *vimA* mutants and strain HG66, which possess O-LPS but lack A-LPS, produce no diffuse HBP35 and RgpB bands on a gel (Fig. 3A and B; Shoji et al. 2011). This result indicates that A-LPS is critical for forming the diffuse bands of HBP35 and RgpB. We have recently shown that the mAb 1B5-immunoreactive products of the PorSS (T9SS)-related mutant cell lysates had lower in molecular masses than those of the wild type (Fig. S3), suggesting that the *P. gingivalis* wild type has abundant glycosylated CTD proteins bound to A-LPS (Shoji et al. 2011). Interestingly, the mAb TDC-5-2-1-immunoreactive products of the PorSS-related mutant cell lysates were also lower in molecular masses than those of the wild type (Fig. S3). Why are mAb TDC-5-2-1-immunoreactive products of the PorSS (T9SS)-related mutants lower in molecular masses than those of the wild type? As it is unlikely that CTD proteins bind to O-LPS, A-LPS may include a glycan epitope that is recognized by mAb TDC-5-2-1. However, saccharides composing APS recognized by mAb 1B5 are not involved in the conventional O-antigen (Paramonov et al. 2001, 2005). To elucidate the source of this contradiction, further analysis is needed to identify the glycan epitope of mAb TDC-5-2-1.

It has been reported that not only CTD proteins but also OMP85 (Nakao et al. 2008) and Mfa1 (Zeituni et al. 2010) proteins are glycosylated in *P. gingivalis*. As the latter two proteins present discrete bands, the glycosylation mechanism of those proteins may be different from that of CTD proteins. In other bacteria, N-linked or O-linked protein glycosylation mechanisms are reported, and these protein glycosylation mechanisms are mediated by specific oligosaccharyltransferases (Hug and Feldman 2011). It has been shown that *Campylobacter jejuni* PglB has dual functions as an N-linked oligosaccharyl transferase and an O-antigen ligase (WaaL) (Feldman et al. 2005), and *Burkholderia thailandensis* PglL_Bt_ and *V. cholera* PglL_Vc_ have an O-linked oligosaccharyl transferase and contain a Wzy_C domain that is also present in WaaL (Gebhart et al. 2012). In *P. gingivalis*, the best-matched Wzy_C domain was found in PGN_1302 (WaaL) (Rangarajan et al. 2008). Further analysis is needed to determine whether PGN_1302 has protein glycosylation activity. PorSS-related genes are conserved in the *Bacteroidetes* phylum but are lacking in gut bacteria such as *B. fragilis* and *Bacteroides thetaiotaomicron* (Sato et al. 2010). We found that other bacteria within the *Bacteroidetes* phylum lack some A-LPS biosynthesis-related genes present in *P. gingivalis* (Table S1). MAb 1B5 does not recognize the cell lysates of *Prevotella intermedia*, *Tannerella forsythia*, and *Flavobacterium johnsoniae* (data not shown). Therefore, the mechanism of A-LPS biosynthesis and the glycosylation mechanism of CTD proteins may be specific to *P. gingivalis* among members of the *Bacteroidetes* phylum. As the glycosylation mechanism provides the robust pigmentation, gingipain activities, and hemagglutinin activity (Figs. 1B and 2A and B), it is important to identify this unknown mechanism. As shown in Figure 1, the biosynthesis pathway of the two LPSs is undertaken by WbaP homologs (PGN_1896 and PGN_1233), Wzx homolog (PGN_1033), Wzy homolog (PGN_1242), WzzP homolog (PGN_2005), and WaaL homolog (PGN_1302). LPS biosynthetic pathways are classified into four types: *Salmonella enterica* LPS, *P. aeruginosa* LPS, *E. coli* LPS, and *Helicobacter pylori* LPS (Hug and Feldman 2011). The LPS biosynthetic pathway of *P. gingivalis* is similar to that of *S. enterica*. A-LPS and O-LPS are present on the cell surface in the PorSS (T9SS)-related mutants (Shoji et al. 2011; data not shown), and the *waaL* mutant is PorSS (T9SS)-proficient because it demonstrated hemolysis activity on a blood agar plate (Rangarajan et al. 2008), suggesting that the LPS transport pathway is independent from PorSS (T9SS) (Fig. 11).

In conclusion, we found a novel *P. gingivalis* pigment-less mutant by transposon mutagenesis: the gene responsible was the PGN_2005 gene of strain ATCC 33277. On the basis of analysis of the LPS pattern of the PGN_2005 mutant, the location and structure of the PGN_2005 protein and complementation of an *E. coli wzz* mutant by the PGN_2005 gene, we believe that the PGN_2005 protein is Wzz of *P. gingivalis*, designated WzzP. In addition, in this study, we determined that the PGN_1896 and PGN_1233 proteins and the PGN_1033 protein appear to be WbaP homolog proteins and a Wzx homolog protein, respectively.

## Supporting Information

**Figure S1.** Multiple sequence alignments of PGN_2005 homolog proteins. Amino acid sequences of PGN_2005 from *Porphyromonas gingivalis*, Poras_0604 from *P. asaccharolytica*, PORUE0001_0693 from *P. uenosis*, and POREN0001_1785 from *P. endodontalis* were compared. Red- or green-colored amino acids indicate putative transmembrane regions shown as TM1 and TM2, respectively (as judged by the SOSUI program).

**Figure S2.** Phylogenetic tree of the amino acid sequences of the Wzz family. The phylogenetic tree was constructed using concatenated amino acid sequences (A) or whole amino acid sequences (B). The scale bar indicates amino acid substitutions per site. Each number of amino acid sequences used for analysis is depicted.

**Figure S3.** Immunoblot analyses of various *Porphyromonas gingivalis* PorSS-related deficient mutants or of various purified LPSs. The cell lysates of various *P. gingivalis* PorSS-related deficient mutants were subjected to SDS-PAGE, and immunoblot analyses were performed with mAb 1B5 and mAb TDC-5-2-1 (A). The purified LPS fractions (using the Intron kit) from various strains were detected with mAb 1B5 or mAb TDC-5-2-1, as appropriate (B).

**Data S1.** Experimental procedures.

**Table S1.** Comparative genome analyses of A-LPS biosynthesis related genes.

**Table S2.** Bacterial strains used in this study.

**Table S3.** Plasmids used in this study.

**Table S4.** Oligonucleotides used in this study.
