# Supplementary material for: Identification of an O-antigen chain length regulator, WzzP, in Porphyromonas gingivalis
Source: Microbiologyopen. 2013 Mar 19;2(3):383–401. doi: 10.1002/mbo3.84 (PMC3684754; doi:10.1002/mbo3.84)
Supplement: Supplementary file 1 [file mbo30002-0383-SD1.doc]

**Data S1. Experimental procedures.**

**Construction of *P. gingivalis* mutants**

To create a *P. gingivalis* PGN_2005::Em^r^ mutant (KDP206), the upstream region of PGN_2005 was amplified with PGN_2005upFw/PGN_2005upBw and was cloned into the pGEM-T Easy vector. The downstream region of PGN_2005 was amplified with PGN_2005dwFw/PGN_2005dwBw and was cloned into the pGEM-T Easy vector. Each upstream and downstream region was inserted into the appropriate restriction region of pBluescript II SK(-). Then, a BamHI-BamHI fragment containing an *ermF* gene was inserted into the BamHI site of the recombinant plasmid to yield pKD870. pKD870 linearised with NotI was introduced into a *P. gingivalis* ATCC 33277 by electroporation and was then selected on blood agar plates containing Em (10 μg/ mL).

To create a *P. gingivalis* PGN_2005::Em^r^ *porT*::Ap^r^ mutant (KDP207), the upstream region of *porT* was amplified with PorTupFw/PorTupBw and cloned into the pGEM-T Easy vector. The downstream region of *porT* was amplified with PorTdwFw/PorTdwBw and was cloned into the pGEM-T Easy vector. Each upstream and downstream region was inserted into the appropriate restriction region of pBluescript II SK(-). Then, a BamHI-BamHI fragment containing a *cepA* gene was inserted into the BamHI site of the recombinant plasmid to yield pKD871. pKD871 linearised with BssHII was introduced into a *P. gingivalis* PGN_2005::Em^r^ mutant (KDP206) and was then selected on blood agar plates containing Ap (10 μg/ mL).

To create *P. gingivalis* PGN_1033::Em^r^ (KDP208), PGN_1916-1917::Em^r^ (KDP209), PGN_2066::Em^r^ (KDP210), PGN_2072::Em^r^ (KDP211), PGN_1523-1525::Em^r^ (KDP212), PGN_1362-1363::Em^r^ (KDP213), PGN_1896::Em^r^ (KDP214) and PGN_0223-0227::Em^r^ (KDP215) mutants, the upstream regions were amplified with PGN_1033upFw/PGN_1033upBw, PGN_1917upFw/PGN_1917upBw, PGN_2066upFw/PGN_2066upBw, PGN_2072upFw/PGN_2072upBw, PGN_1523upFw/PGN_1523upBw, PGN_1362upFw/PGN_1362upBw, PGN_1896upFw/PGN_1896upBw and PGN_0223upFw/PGN_0223upBw, respectively, and cloned into the pCR4 vector or the pGEM-T Easy vector. The downstream region was amplified with PGN_1033dwFw/PGN_1033dwBw, PGN_1916dwFw/PGN_1916dwBw, PGN_2066dwFw/PGN_2066dwBw, PGN_2072dwFw/PGN_2072dwBw, PGN_1525dwFw/PGN_1525dwBw, PGN_1363dwFw/PGN_1363dwBw, PGN_1896dwFw/PGN_1896dwBw and PGN_0227dwFw/PGN_0227dwBw, respectively, and cloned into the pCR4 vector. Each upstream and downstream region was swapped with the appropriate restriction region of pKD740, giving rise to from pKD872 to pKD879, respectively. The resulting targeting vector, linearised with SacI, was introduced into *P. gingivalis* ATCC 33277 by electroporation and was then selected on blood agar plates containing Em (10 μg/ mL).

To create a *P. gingivalis* PGN_1233::Tc^r^ (KDP216) mutant, a BamHI-BglII fragment containing a *tetQ* gene from pKD375 (Shi *et al*., 1999) was inserted into the BamHI site of pBluescript II SK(-) to yield pKD880. Next, a BamHI-PstI fragment containing a *tetQ* gene derived from pKD880 was inserted into the same region of pKD740 (Shoji *et* *al*., 2010) to yield pKD881. The upstream region of PGN_1233 was amplified with PGN_1233upFw/PGN_1233upBw and cloned into the pCR4 vector. The downstream region of PGN_1233 was amplified with PGN_1233dwFw/PGN_1233dwBw and cloned into the pCR4 vector. Each upstream and downstream region was swapped with the appropriate restriction region of pKD881. The resulting targeting vector pKD882, linearised with SphI, was introduced into *P. gingivalis* ATCC 33277 by electroporation and was then selected on blood agar plates containing Tc (0.7 μg/ mL).

To create a *P. gingivalis* PGN_1233::Tc^r^ PGN_1896::Em^r^ double mutant (KDP217), pKD878, linearised with SacI, was introduced into a *P. gingivalis* PGN_1233::Tc^r^ mutant (KDP216) by electroporation and was then selected on blood agar plates containing Em (10 μg/ mL).

**Construction of *P. gingivalis* complemented strains**

To create complemented strains, a DNA fragment containing the PGN_2005 gene was amplified with PGN_2005compFw/PGN_2005compBw and cloned into pGEM-T Easy vector to yield pKD883. The promoter region of the *Porphyromonas gulae* *catalase* gene (accession no. AB083039 in GenBank/EMBL/DDBJ databases) was amplified with P6-34-F-NotI/ P6-34-R-SalI from pKD955 (Sato *et al*., 2010) and cloned into the pGEM-T easy vector to yield pKD884. A SalI DNA fragment containing the PGN_2005 gene of pKD883 was inserted into the SalI site of pKD884 to yield pKD885. The terminator region of *rgpB* gene was amplified with 506-TF-PstI/506-TR-NotI from pKD955 (Sato *et al*., 2010) and was digested with PstI and NotI, prior to insertion in the same region of pBSSK to yield pKD886. The EcoRI-PstI DNA fragment from pKD885 containing the promoter region of the *catalase* gene and the PGN_2005 gene was inserted into the same region of pKD886 to yield pKD887. The NotI DNA fragment of pKD887 was inserted into the NotI site of a pTCB vector to yield pKD888. pKD888 was introduced into *Escherichia coli* S17-1 by electroporation and was then selected on LB agar plates containing Ap (100 μg/ mL). The transformant was mated with a PGN_2005::Em^r^ mutant (KDP206) or a PGN_2005::Em^r^ *porT*::Ap^r^ mutant (KDP207) and was then selected on blood agar plates containing Gm (50 μg/ mL) and Tc (0.7 μg /mL), yielding PGN_2005::Em^r^/pKD888 (KDP218) and PGN_2005::Em^r^ *porT*::Ap^r^/pKD888 (KDP219).

**Preparation of mAb TDC-5-2-1**

mAb TDC-5-2-1 was made previously (Maruyama *et al*., 2009). Detailed description of preparation of mAb TDC-5-2-1 is as follows. A bacterial secreted antigen of *P. gingivalis* TDC60 was used to generate mouse mAb TDC-5-2-1. The secreted antigen was precipitated using a 40% NH_2_SO_4_ saturation from bacterial cell culture medium of *P. gingivalis* TDC60, and the precipitate was then collected by centrifugation at 20,000 g for 40 min. Finally, the pellet was suspended in PBS, pH 7.2, and dialysed against the same buffer. Three mice were immunised with the secreted antigen. Splenocytes from the immunised mice were harvested and fused with a mouse myeloma cell line using standard techniques. Cell culture supernatants from wells containing hybridoma colonies were then used to determine the level of binding with the secreted antigen preparation in an enzyme-linked immunosolvent assay (ELISA). All cells from the positive wells were expanded and retested. Cultures that remained positive were sub-cloned to generate stable, clonal hybridoma cell lines expressing mAbs reactive to the secreted antigen preparation from *P. gingivalis* TDC60. Finally, the cell culture supernatants, including the mAb TDC 5-2-1, were purified using a protein G column.
