# Supplementary material for: Identification of an O-antigen chain length regulator, WzzP, in Porphyromonas gingivalis
Source: Microbiologyopen. 2013 Mar 19;2(3):383–401. doi: 10.1002/mbo3.84 (PMC3684754; doi:10.1002/mbo3.84)

# Supplemental 1

```
                                                           TM1
Poras_0604            MTQSSLPNRAVYNEPTRSDEGTRPTFSQILRLILKQWYWFVLSFVLFVVLGKIVADHRPL   60
PORUE0001_0693        MTQSSLPNSAVNDQPNYPDEGSRPTFSQILRLILRQWYWFVIFIAIFVWLGCSIVKYRPL   60
POREN0001_1785        MDSTFSPSKTVLP--------QRMSFSAFVRLVVLNWYWFALSLVVAFGFIKMFGNSSAI   52
PGN_2005              MTEKSFRSVVLPK---------DSVLTLFFRICLRNWYWILISVVLFAGWGVVKYKRAGM   51
                      * ..   . .:              :: :.*: : :***: : ..:         .   :

Poras_0604            GRQTYYMQLSSREPHELTNPMMTLSDS-----------TFKTSPWTPEYIATLLNTTTAV  109
PORUE0001_0693        GRQTYYMQLSSREPHEPTEPTMQLGDP-----------IPKTSPWAPEYIASLLNTTTAV  109
POREN0001_1785        GNNKYRMAVMTRFPIQTSKSDKVFVDANS---GKEAPLWKHTPAFDAAQIYGWIVSADII  109
PGN_2005              APSTYFTTVMARFPEGVQRASSGGLNPNYNVYDNYTPRWEGISVLDQNSIFNGLCATSLV  111
                      . ..*   : :* *    ..     :.               .      *   : ::  :

Poras_0604            VEAGRRINFQVDYFVKTPWGMRDYYNQTPIIVHFDDLLPTDKVHCIAKLDDPVHPQQVTL  169
PORUE0001_0693        LEAGKRIDFQVDYWVKTPFGMRDYYNQTPIIVRFGNLLPTDKLYCIARLDDPVHPKEVTM  169
POREN0001_1785        YRTGKREGFEVEYAQRSGFSSRDIYNHIPFRLIFPDAADADYFTLVVRQEA----DGLQL  165
PGN_2005              ERVGEKIQYDVNYYLPGKVTNHDFYDHTPIHLIFSPESALSSFTVKMKVEK----EHATI  167
                       ..*.:  ::*:*        :* *:: *: : *      . .    : :     .   :

Poras_0604            SGFLGTAQGISINEP--YDVVIPVGSTQETPVGHVSITLDNPKHHFPYHIDQKHKKIALS  227
PORUE0001_0693        SAFSGVVQGQPLYEP--YDVVIPVGTTQETPVGPITVTLDDPAQHFPYHIDQQYTEIPIT  227
POREN0001_1785        SQIKGVYQGKELKGTGSYTILLPWNSTVETPVGKVILQDNPGWEEYGIRSYALDKPIYIT  225
PGN_2005              LSLKGKHDGEKVSIK---DIRLPYGISVATPIGELRVEKTPRFDYETSKKDFFSKPIIIV  224
                        : *  :*  :       : :* . :  **:* : :      .    :     . * :

Poras_0604            YS-TLQDIRAVYETEMQVTMKKADLPLSSVMRVEMLSNRSERRCLDLLYQMYLVTDSLGW  286
PORUE0001_0693        YT-SLVEARDIYETEMQVTMKKADLPLSSVMRVELLSGRSERRCLSLLNAMYVVTDSLGW  286
POREN0001_1785        RR-SVIETRAQYDWDMVIDN-----TLPNFLNVEVSTTGSARRAKQILAGMLVDLDSLVR  279
PGN_2005              TKESKDKARVLYDSGLRTEE------SKGRIEMRITIKRSLGFARDFLDGIIEEFDIQSR  278
                         :  . *  *:  :            . :.:.:    *   . .:*  :    *

Poras_0604            VEQLSDEGVTDSLGQFAG--TVPPAGLSP--------FRLIDEPRIAKDLEPDLFIIAG-  335
PORUE0001_0693        VEQLTDEGHVDSLGQFVG--TVPPTGLSP--------FRLVDKPRELREIEPDLFVIVG-  335
POREN0001_1785        YNVTLDLDKEQEMIEAALRRLMHPDSLSPS-------LTAESRTQEIKELQQRLARTISN  332
PGN_2005              EKYRQEVEEKIALAEKSLSDLQKSEGPVSDSTLVSLGITEKERNADSEALRKKLVSMINE  338
                       :   :      : :        . .  .        :   ..    . :.  *    .
                                                                 TM2
Poras_0604            -----------------------------------MGLLGLLLPLLLMYIYWAIRGVIYY  360
PORUE0001_0693        -----------------------------------MGLLGFLIPLLLLYIYWAILGAIYY  360
POREN0001_1785        REVLRYDHLIE----VTASPAIQPSPVGAFSVKIIFLLLGLIIPTVLIYYAWLLRGSVLE  388
PGN_2005              YKVDLLTADASSSLIVLDKTKSVPVSKKDLLILIINLVMGLFFPMIIFFVYIVFRNLILV  398
                                                           ::*:::* ::::    : . :

Poras_0604            VSELPSLLRHRLT--LDLKRGRKGKKPLYPNLEELCALVMPP---QGEPRTIQLATPNST  415
PORUE0001_0693        VCELPRLLRSRLT--LDLQRRHKG-KTLYPHLEELCALVSP----QGEPRAIQLVTPSGT  413
POREN0001_1785        PRQLSPFWQKALISTLRLHRKRGNYSDTPETIDALRFTLQSNLEEDTDARPILFTTPSKS  448
PGN_2005              PSELGEKALKRILALLNGNKKRAATMQRDIDLLRLNLTEYLP---DSASSCIILTGMSGN  455
                        :*       :   *  :: :         :  *          :  .  * :.  . .

Poras_0604            KYHDQLLAELQQALEQRGISCAVWHLDLAEQKAVSKT--PSDVYHTTLTPGLISSTAFQQ  473
PORUE0001_0693        KYHRQLASELQQALQQRGISCAVWYLDLANQKKASKG--SNGVYHTTITPGLIGSSAFEQ  471
POREN0001_1785        SKAQKRLNKLVADLAASYSLGGGNTGKIVHLLHASNNSVPTLSHARIIEQGYWGGKQFSQ  508
PGN_2005              EQTERIATELVSSFERK--------GKNFQILHLEES-----------------DAALK  489
                      .   :   :*   :            .  .    .:                       :

Poras_0604            QLKQLQTQHQLTIIASPALMEDPTAYLLSSSVEQSLYCIYRGSTRVRAIKRIVHQLQIRQ  533
PORUE0001_0693        QLKQLQTQHQLTIIVPPALTKDPTAYLLN-SLEQSLYCIYRGSTRIGAIKRVVHQLQIRQ  530
POREN0001_1785        DLAPAEAERKTPTLLIAPFDS---VHLLASQVAQIVVVVEQDQSRTTLLSDLEHSLSQGT  565
PGN_2005              EMTALREKGISVFAIAPSVVRSALAVEVSFVADVSLLLAESMHSKRNDKDEVEAFICQSR  549
                      ::   . :        ...     .  :       :       ::    . :   :

Poras_0604            LLDRSRIEILWIE------- 546
PORUE0001_0693        QLDRSHIHTLWVE------- 543
POREN0001_1785        PP----IHALWVCNIFPFFR 581
PGN_2005              CP----AFVLWLDRY----- 560
                               **:
```

Supplemental 3

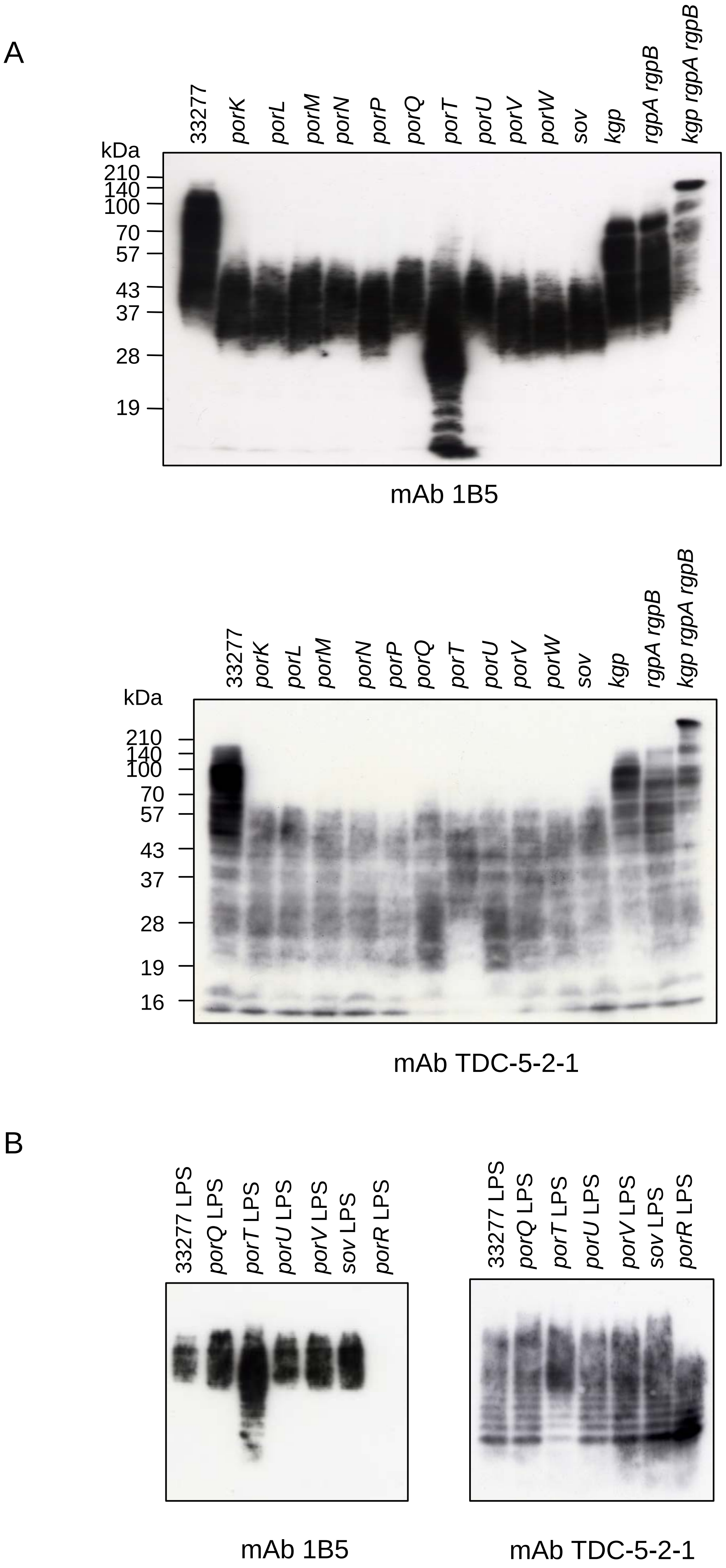

Supplement: Supplementary file 2 [file mbo30002-0383-SD2.pdf]
