# Supplementary figures and images for: Identification of an O-antigen chain length regulator, WzzP, in Porphyromonas gingivalis
Source: Microbiologyopen. 2013 Mar 19;2(3):383–401. doi: 10.1002/mbo3.84 (PMC3684754; doi:10.1002/mbo3.84)

Supplemental 2

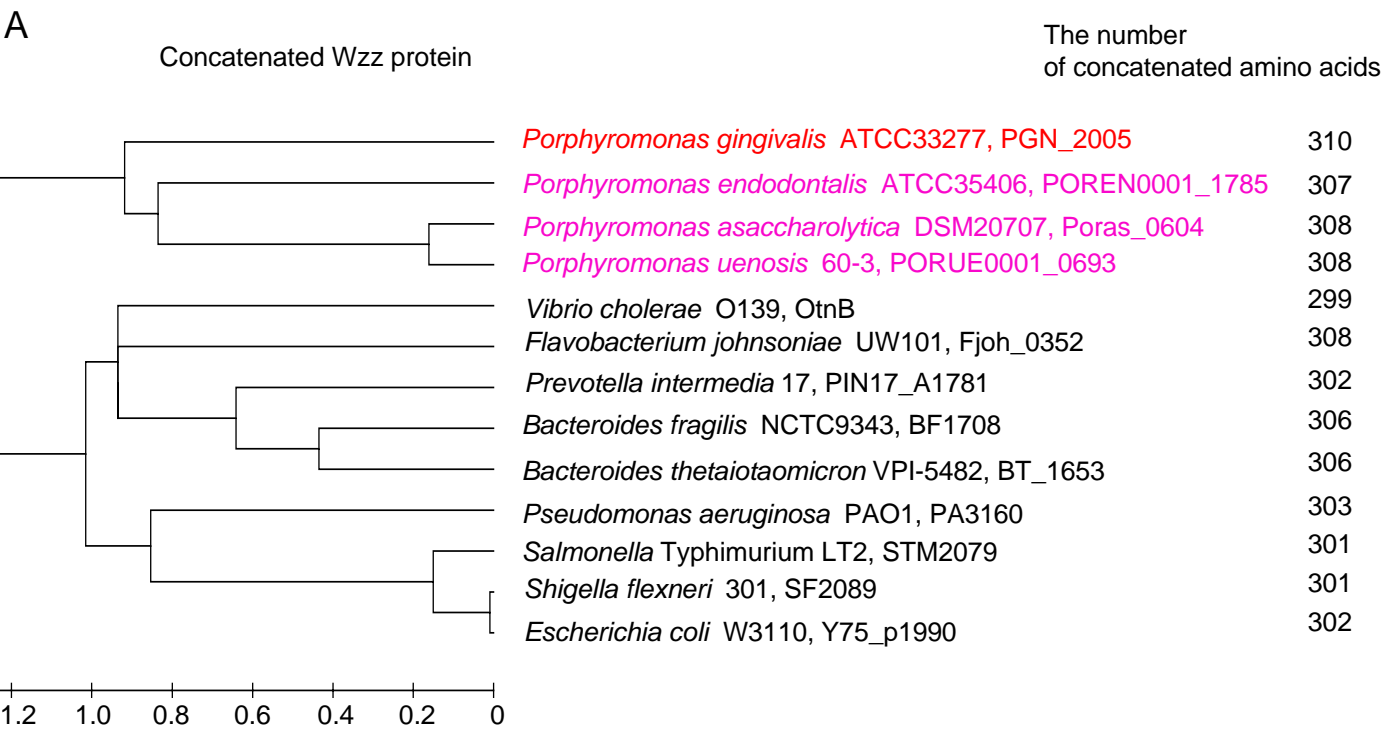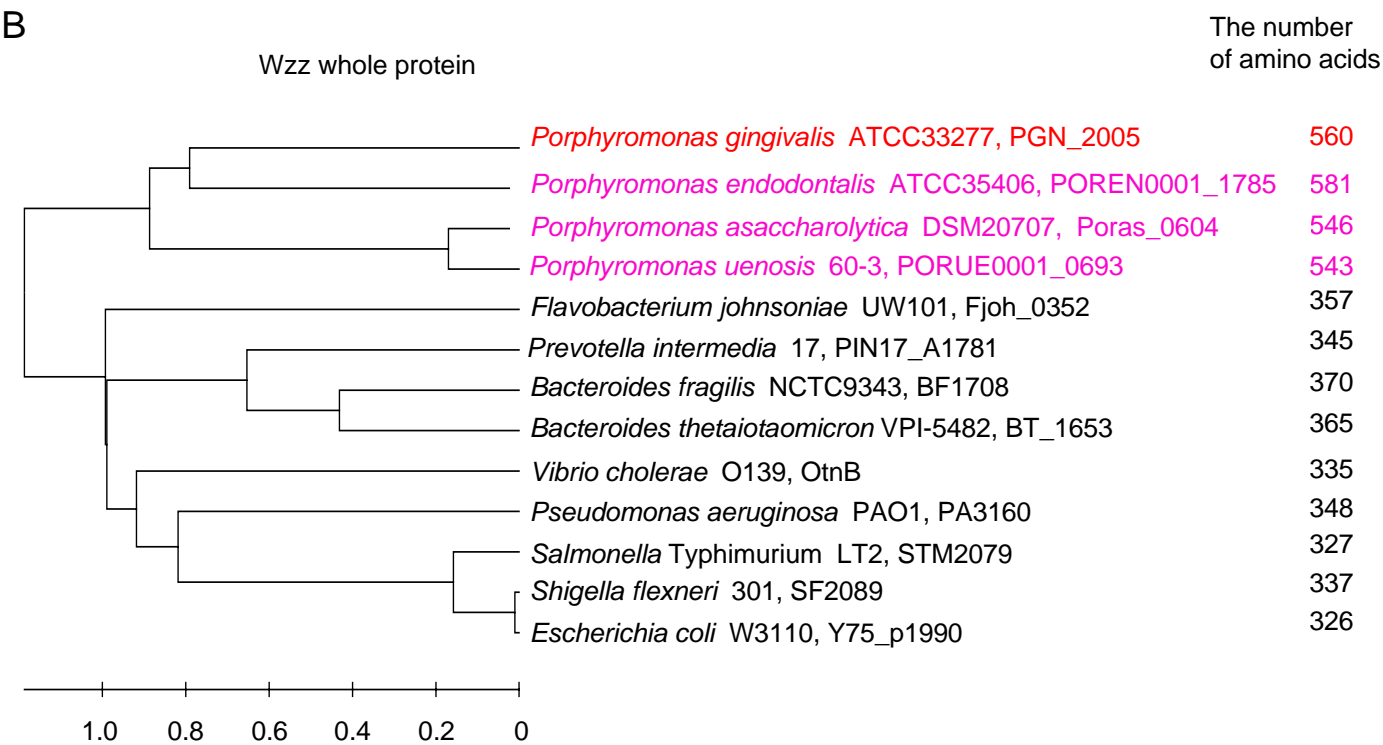

Supplement: Supplementary file 3 [file mbo30002-0383-SD3.pdf]
