# Supplementary material for: Identification of an O-antigen chain length regulator, WzzP, in Porphyromonas gingivalis
Source: Microbiologyopen. 2013 Mar 19;2(3):383–401. doi: 10.1002/mbo3.84 (PMC3684754; doi:10.1002/mbo3.84)
Supplement: Supplementary file 4 [file mbo30002-0383-SD4.pdf]

Table S1. Comparative genome analyses of A-LPS biosynthesis related genes.

Strain	*man*	*hypothetical*	*vimA*	*porR*
P. gin ATCC33277	PGN_0242	PGN_0663	PGN_1056	PGN_1236
P. gin W83	PG0129	PG0621	PG0882	PG1138
P. gin TDC60	PGTDC60_0406	PGTDC60_1749	PGTDC60_0810	PGTDC60_1075
P. asa DSM20707	Poras_1033	Poras_1142	Poras_0672	Poras_0235<br>Poras_0456<br>Poras_0968
P. uenosis 60-3	PORUE0001_0304<br>PORUE0001_1218	PORUE0001_1022	PORUE0001_1328	PORUE0001_0620<br>PORUE0001_1509
P. endo ATCC35406	POREN0001_0178	POREN0001_0416	POREN0001_1221	POREN0001_0099<br>POREN0001_0534
P. int 17	PIN17_A1289	PIN17_A1908	ND	PIN17_A1561<br>PIN17_A1764
T. for ATCC43037	BFO_0475	BFO_2611	BFO_2568	BFO_0923<br>BFO_1073
F. joh UW101	Fjoh_0326<br>Fjoh_1035<br>Fjoh_2513	Fjoh_3498	ND	Fjoh_0323<br>Fjoh_0334<br>Fjoh_1727<br>Fjoh_2237
B. fragilis NCTC9343	BF0920	BF3731	ND	BF0734<br>BF0752<br>BF1376<br>BF2604
B. the VPI-5482	BT_4282<br>BT_4304	BT_3927	ND	BT_0612<br>BT_2885<br>BT_3376

Strain	*wzy*	*gtfB*	*rfa*	*waaL*
P. gin ATCC33277	PGN_1242	PGN_1251	PGN_1255	PGN_1302
P. gin W83	PG1142	PG1149	PG1155	PG1051
P. gin TDC60	PGTDC60_1070	PGTDC60_1060	PGTDC60_1065	PGTDC60_0972
P. asa DSM20707	Poras_0568	Poras_1033<br>Poras_1145<br>Poras_1514	ND	Poras_0494
P. uenosis 60-3	PORUE0001_1456	PORUE0001_0103	ND	PORUE0001_1746
P. endo ATCC35406	POREN0001_1267	ND	POREN0001_1404	POREN0001_0324
P. int 17	PIN17_A1783	PIN17_A1552	PIN17_A0550	ND
T. for ATCC43037	ND	BFO_1049	BFO_1088	ND
F. joh UW101	Fjoh_2813	Fjoh_0341<br>Fjoh_2243	Fjoh_1046	Fjoh_0291
B. fragilis NCTC9343	BF1018<br>BF3456<br>BF3686	BF2052<br>BF2595	ND	ND
B. the VPI-5482	BT_1343<br>BT_1710	BT_0053<br>BT_1180<br>BT_2866	BT_3362	ND

Strain	*wzz*	*wzx*	*wbaP*
P. gin ATCC33277	PGN_2005	PGN_1033	PGN_1896
P. gin W83	PG0056	PG0912<br>PG0117	PG1964
P. gin TDC60	PGTDC60_0338	PGTDC60_0836	PGTDC60_0237
P. asa DSM20707	Poras_0604	Poras_0962	Poras_0564
P. uenosis 60-3	PORUE0001_0693	PORUE0001_0631	PORUE0001_0240
P. endo ATCC35406	POREN0001_1785	POREN0001_1270	POREN0001_0361
P. int 17	PIN17_A1781	PIN17_A1782	PIN17_A1560<br>PIN17_A1779
T. for ATCC43037	ND	BFO_1985	BFO_1803<br>BFO_3047
F. joh UW101	Fjoh_0352	Fjoh_0345	Fjoh_4994
B. fragilis NCTC9343	BF1708	BF1369<br>BF1556<br>BF1900<br>BF2055<br>BF2598<br>BF2791<br>BF4298	BF1377
B. the VPI-5482	BT_1355<br>BT_1653<br>BT_1722	BT_0040<br>BT_0047<br>BT_1354	BT_0480

man, putative mannosyl transferase
ND, not detected

P. gin, *Porphyromonas gingivalis* ATCC33277, W83 or TDC60
P. asa, *Porphyromonas asaccharolytica* DSM20707
P. uenosis, *Porphyromonas uenosis* 60-3
P. endo, *Porphyromonas endodontalis* ATCC35406
P. int, *Prevotella intermedia* 17
T. for, *Tannerella forsythia* ATCC43037
F. joh, *Flavobacterium johnsoniae* UW101
B. fragilis, *Bacteroides fragilis* NCTC9343
B. the, *Bacteroides thetaiotaomicron* VPI-5482
