# Supplementary material for: Identification of an O-antigen chain length regulator, WzzP, in Porphyromonas gingivalis
Source: Microbiologyopen. 2013 Mar 19;2(3):383–401. doi: 10.1002/mbo3.84 (PMC3684754; doi:10.1002/mbo3.84)
Supplement: Supplementary file 5 [file mbo30002-0383-SD5.pdf]

Table S2. Bacterial strains used in this study.

P. gingivalis strains	Description	Reference or source
33277	Wild type	ATCC
KDP107	*porR* ::Em^r	Shoji et al. 2002
KDP117	*porT* ::Em^r	Sato et al. 2005
KDP129	*kgp::* Cm^r	Shi et al. 1999
KDP133	*rgpA::* Em^r *rgpB::* Tc^r	Shi et al. 1999
KDP136	*kgp* ::Cm^r *rgpA* ::Em^r *rgpB* ::Tc^r	Shi et al. 1999
KDP200	PGN_0242::Em^r	Shoji et al. 2011
KDP201	PGN_0663::Em^r	Shoji et al. 2011
KDP202	*vimA* ::Tc^r	Shoji et al. 2011
KDP203	*waaL* ::Em^r	Shoji et al. 2011
KDP204	*wzy* ::Em^r	Shoji et al. 2011
KDP205	PGN_2005::Tn*4400'* , Tc^r	This study
KDP206	PGN_2005::Em^r	This study
KDP207	PGN_2005::Em^r *porT* ::Ap^r	This study
KDP208	PGN_1033::Em^r	This study
KDP209	PGN_1916-1917::Em^r	This study
KDP210	PGN_2066::Em^r	This study
KDP211	PGN_2072::Em^r	This study
KDP212	PGN_1523-1525::Em^r	This study
KDP213	PGN_1362-1363::Em^r	This study
KDP214	PGN_1896::Em^r	This study
KDP215	PGN_0223-0227::Em^r	This study
KDP216	PGN_1233::Tc^r	This study
KDP217	PGN_1233::Tc^r PGN_1896::Em^r	This study
KDP218	PGN_2005::Em^r/pKD888	This study
KDP219	PGN_2005::Em^r *porT* ::Ap^r/pKD888	This study
KDP354	*porP* ::Em^r	Sato et al. 2010
KDP355	*porK* ::Em^r	Sato et al. 2010
KDP356	*porL* ::Em^r	Sato et al. 2010
KDP357	*porM* ::Em^r	Sato et al. 2010
KDP358	*porN* ::Em^r	Sato et al. 2010
KDP359	*porW* ::Em^r	Sato et al. 2010
KDP360	*porU* ::Em^r	Sato et al. 2010
KDP361	*porV* ::Em^r	Shoji et al. 2011
KDP362	*porQ* ::Em^r	Sato et al. 2010
KDP365	*sov* ::Em^r	Sato et al. 2010
KDP400	*gtfB* ::Em^r	Yamaguchi et al. 2010
Prfa1	*rfa* ::Em^r	Sato et al. 2009
W83	Wild type	Gift from M. J. Duncan[a]
TDC60	Wild type	Gift from K. Ishihara[b]
TDC117	Wild type	Gift from K. Ishihara[b]
TDC275	Wild type	Gift from K. Ishihara[b]
GAI7802	Wild type	Gift from E. Hoshino[c]
SU63	Wild type	Gift from M. Yoneda[d]
HG66	non pigmented strain	Gift from J. Potempa[e]

E. coli strains	Description	Source or reference
XL-1Blue	General purpose host strain for cloning	Stratagene
BL21(DE3)	Host strain for protein expression	Novagen
S17-1	RP-4-2-Tc::Mu *aph* ::Tn7 *recA* , Sm^r	Simon et al. 1983
W3110	*rph-1* 1N(*rrnD-rrnE*)	Gift from M. A. Valvano[f]
EVV16	W3110 Δ*wzzB* ::Km^r	Vinés et al. 2005

a Department of Molecular Genetics, The Forsyth Institute, Boston, MA.
b Department of Microbiology, Tokyo Dental College, Chiba, Japan.
c Department of Oral Health Science, Graduate School of Medical and Dental Sciences, Niigata University, Niigata, Japan.
d Department of General Dentistry, Fukuoka Dental College, Fukuoka, Japan.
e Faculty of Biochemistry Biophysics and Biotechnology, Jagiellonian University, Krakow, Poland.
f Department of Microbiology and Immunology, University of Western Ontario, Ontario, Canada.
