# Supplementary material for: Identification of an O-antigen chain length regulator, WzzP, in Porphyromonas gingivalis
Source: Microbiologyopen. 2013 Mar 19;2(3):383–401. doi: 10.1002/mbo3.84 (PMC3684754; doi:10.1002/mbo3.84)
Supplement: Supplementary file 6 [file mbo30002-0383-SD6.pdf]

Table S3. Plasmids used in this study.

Plasmids	Description	Source or reference
pBAD/Myc- His A	Ap^r, arabinose indusible expresison plasmid	Invitrogen
pBluescript II SK(-)	Ap^r, cloning vector	Stratagene
pCR4	Ap^r Km^r, PCR Topo cloning vector	Invitrogen
pET30 Ek/LIC	Km^r, IPTG indusible expresison plasmid	Novagen
pGEM-T Easy	Ap^r, PCR cloning vector	Promega
pKD375	Ap^r Tc^r, pUC19-*tetQ*	Shi et al., 1999
pKD740	Ap^r Em^r, pGEM-T Easy-*hbp35* up-*ermF* -*hbp35* dw	Shoji et al., 2010
pKD870	Ap^r Em^r, pBSSK-PGN_2005up-*ermF* -PGN_2005dw	This study
pKD871	Ap^r, pBSSK-*porT* up-*cepA* -*porT* dw	This study
pKD872	Ap^r Em^r, pGEM-T Easy-PGN_1033up-*ermF* -PGN_1033dw	This study
pKD873	Ap^r Em^r, pGEM-T Easy-PGN_1917up-*ermF* -PGN_1916dw	This study
pKD874	Ap^r Em^r, pGEM-T Easy-*PGN_* 2066up-*ermF* -PGN_2066dw	This study
pKD875	Ap^r Em^r, pGEM-T Easy-PGN_2072up-*ermF* -PGN_2072dw	This study
pKD876	Ap^r Em^r, pGEM-T Easy-PGN_1523up-*ermF* -PGN_1525dw	This study
pKD877	Ap^r Em^r, pGEM-T Easy-PGN_1362up-*ermF* -PGN_1363dw	This study
pKD878	Ap^r Em^r, pGEM-T Easy-PGN_1896up-*ermF* -PGN_1896dw	This study
pKD879	Ap^r Em^r, pGEM-T Easy-PGN_0223up-*ermF* -PGN_0227dw	This study
pKD880	Ap^r Tc^r, pBSSK-*tetQ*	This study
pKD881	Ap^r Tc^r, pGEM-T Easy-*hbp35* up-*tetQ* -*hbp35* dw	This study
pKD882	Ap^r Tc^r, pGEM-T Easy-PGN_1233up-*tetQ* -PGN_1233dw	This study
pKD883	Ap^r, pGEM-T Easy-PGN_2005	This study
pKD884	Ap^r, pGEM-T Easy-*catalase* pro	This study
pKD885	Ap^r, pGEM-T Easy-*catalase* pro-PGN_2005	This study
pKD886	Ap^r, pBSSK-*rgpB* ter	This study
pKD887	Ap^r, pBSSK-*catalase* pro-PGN_2005-*rgpB* ter	This study
pKD888	Ap^r Tc^r, pTCB-*catalase* pro-PGN_2005-*rgpB* ter	This study
pKD889	Km^r, pET30 Ek/LIC PGN_2005 [L^{395}-Y^{560}]	This study
pKD890	Ap^r, pUC118- PGN_2005	This study
pKD891	Ap^r, pBAD/Myc-His A-PGN_2005	This study
pKD955	Ap^r, pBSSK-*catalase* pro-*porK* -*rgpB* ter	Sato et al., 2010
pMF19	Sp^r, pEXT21-*wbbL* (*rhamnosyltransferase*)	Feldman et al., 1999
pTCB	Ap^r, Tc^r, *E. coli* -*P. gingivalis* shuttle plasmid	Nagano et al., 2007
pUC118	Ap^r, cloning vector	Takara
pWzzB-SF	Ap^r, pBAD24-*wzzB* $_{SF}$	Kalynych et al., 2011
pWzzB-ST	Ap^r, pBAD24-*wzzB* $_{ST}$	Kalynych et al., 2011
