# Supplementary material for: Identification of an O-antigen chain length regulator, WzzP, in Porphyromonas gingivalis
Source: Microbiologyopen. 2013 Mar 19;2(3):383–401. doi: 10.1002/mbo3.84 (PMC3684754; doi:10.1002/mbo3.84)
Supplement: Supplementary file 7 [file mbo30002-0383-SD7.pdf]

Table S4. Oligonucleotides used in this study.

Primers	Nucleotide sequences (5'-)	Description of underline
PGN_2005upFw	GCGGCCGCGCTTCTGTACTGAACACGCTG	NotI
PGN_2005upBw	TGGATCCTCGCTCATACTATTAATAGCTG	BamHI
PGN_2005dwFw	AGGATCCTGGTTGGATAGGTATTAAGGGG	BamHI
PGN_2005dwBw	TGGTACCGTTCGATGCTCTCGCATCAACT	KpnI
PorTupFw	TAGGTCGACAGCGCTTGCGGCGGAAAAAGAAG	
PorTupBw	TAGGATCCTAGTTGTCACGCTCTTTTCGAC	BamHI
PorTdwFw	GTGAATTCCCTGAGAGAATAATCTTCAATTCT	EcoRI
PorTdwBw	GCGGCCGCTATACAGGATCGTATTGAGTGCT	NotI
PGN_1033upFw	GCATGCGTTACGAAGTATCGCAATCGCAA	SphI
PGN_1033upBw	GGATCCAACAGACCATAAAGATACTACAA	BamHI
PGN_1033dwFw	CTGCAGAAAGCCTACCTCCATATCCGATT	PstI
PGN_1033dwBw	GAGCTCCACTCGAAGTATGCAGTGGTGTC	SacI
PGN_1917upFw	GCATGCTTGTCGGCGGCGACCTCTCCGTA	SphI
PGN_1917upBw	GGATCCTTGTATTGGTTGTTTCTGTTGTT	BamHI
PGN_1916dwFw	CTGCAGGGAAACGTGAGTTCGATGTAAGC	PstI
PGN_1916dwBw	GAGCTCAGCCGGAGGTGGTATCGGCTCGT	SacI
PGN_2066upFw	GCATGCAAGCAGTTTGCCCTCGCATACTT	SphI
PGN_2066upBw	GGATCCGACTTCGTGCTCATTCGTTTGAT	BamHI
PGN_2066dwFw	CTGCAGGCTCAACCTCCCCCCTCTCTTCT	PstI
PGN_2066dwBw	GAGCTCATCTCCGAAGGTGGCTGTAACCG	SacI
PGN_2072upFw	GCATGCGCTTTGGAGGATACTTTGCCGAT	SphI
PGN_2072upBw	GGATCCTATTATATGCTTGATTCGTATCG	BamHI
PGN_2072dwFw	CTGCAGGCGTCTTTTCGGCCGCAACGAGA	PstI
PGN_2072dwBw	GAGCTCTACATTATGAAGAAATTCATGAT	SacI
PGN_1523upFw	GCATGCCATGAAGCTGCTGCCGTGCCGTC	SphI
PGN_1523upBw	GGATCCAATTGATTTATGGCTTAAAAAAG	BamHI
PGN_1525dwFw	CTGCAGTATCTGACAGATACATTTGTTTG	PstI
PGN_1525dwBw	GAGCTCGCCACTGCTTCTGCGGAACAGCC	SacI
PGN_1362upFw	GCATGCGAGAAGATAGAACGAACTTCTTC	SphI
PGN_1362upBw	GGATCCGAGTTTCTGTCCGGTCGGTCTTT	BamHI
PGN_1363dwFw	CTGCAGACAGAGAGTGAGACGGCACACAG	PstI
PGN_1363dwBw	GAGCTCTGCGTTCGAGATCGAAGTCGGTG	SacI
PGN_1896upFw	GCATGCTACCCGACAGATACCATTTGGGG	SphI
PGN_1896upBw	GGATCCTGGCGGATCACCTCCACTTCTCC	BamHI
PGN_1896dwFw	CTGCAGAGGATAAGGAAACGAGTCGCTAC	PstI
PGN_1896dwBw	GAGCTCGGCCCCACACCCCACGAGGAGCA	SacI
PGN_0223upFw	GCATGCCTCCAATAATCCATCCGGCTACC	SphI
PGN_0223upBw	GGATCCCACAACAATATCTTCAATAACAT	BamHI
PGN_0227dwFw	CTGCAGAAGAAAAAGGTAATGGGAATAGC	PstI
PGN_0227dwBw	GAGCTCGGGAGCTTATGATTTCGAATGAC	SacI
PGN_1233upFw	GCATGCTTTTGTTTTTTTTGCCACTTCCA	SphI
PGN_1233upBw	GGATCCTATCTTCCAATTCAAGGCACTAT	BamHI
PGN_1233dwFw	CTGCAGGTAAAGCTCCGATTGCATAGGGG	PstI
PGN_1233dwBw	GAGCTCGCTACGGAAAGCGTATCTATGAT	SacI
PGN_2005compFw	GTCGACTTGACTGAGAAATCATTTCGAAGT	SalI
PGN_2005compBw	CTGCAGTTAATACCTATCCAACCATAGCAC	PstI
p6-34-F-NotI	GCGGCCGCTTCGTCGTCAATCAGCATCCCAG	NotI
p6-34-R-SalI	GTCGACTGTTTTGTCTCTTATTTAAGTTA	SalI
506-TF-PstI	ACTGCAGTTCACACTGCAATTCTCTAAT	PstI
506-TR-NotI	GCGGCCGCCCTACCCGACCATAAACCGCCA	NotI
PGN_2005expFw	GACGACGACAAGATGCTCATATTGGTACCTTCGGAACTT	
PGN_2005expBw	GAGGAGAAGCCCGGTGCATACCTATCCAACCATAGCACA	
